# Diversity of Mobile Genetic Elements in the Mitogenomes of Closely Related *Fusarium culmorum* and *F. graminearum* sensu stricto Strains and Its Implication for Diagnostic Purposes

**DOI:** 10.3389/fmicb.2020.01002

**Published:** 2020-05-25

**Authors:** Tomasz Kulik, Balazs Brankovics, Anne D. van Diepeningen, Katarzyna Bilska, Maciej Żelechowski, Kamil Myszczyński, Tomasz Molcan, Alexander Stakheev, Sebastian Stenglein, Marco Beyer, Matias Pasquali, Jakub Sawicki, Joanna Wyrȩbek, Anna Baturo-Cieśniewska

**Affiliations:** ^1^Department of Botany and Nature Protection, University of Warmia and Mazury in Olsztyn, Olsztyn, Poland; ^2^Biointeractions & Plant Health, Wageningen Plant Research, Wageningen, Netherlands; ^3^Molecular Biology Laboratory, Institute of Animal Reproduction and Food Research, Polish Academy of Sciences, Olsztyn, Poland; ^4^Department of Animal Anatomy and Physiology, Faculty of Biology and Biotechnology, University of Warmia and Mazury in Olsztyn, Olsztyn, Poland; ^5^Shemyakin and Ovchinnikov Institute of Bioorganic Chemistry, Russian Academy of Sciences, Moscow, Russia; ^6^National Scientific and Technical Research Council, Godoy Cruz, Argentina; ^7^Universidad Nacional del Centro de la Provincia de Buenos Aires, Tandil, Argentina; ^8^Department of Environmental Research and Innovation, Agro-Environmental Systems, Luxembourg Institute of Science and Technology, Belval, Luxembourg; ^9^Department of Food, Environmental and Nutritional Sciences, University of Milan, Milan, Italy; ^10^Laboratory of Phytopathology and Molecular Mycology, Department of Biology and Plant Protection, UTP University of Science and Technology, Bydgoszcz, Poland

**Keywords:** *Fusarium graminearum* sensu stricto, *F. culmorum*, mitogenome, mobile genetic elements, mitochondrial introns, homing endonucleases

## Abstract

Much of the mitogenome variation observed in fungal lineages seems driven by mobile genetic elements (MGEs), which have invaded their genomes throughout evolution. The variation in the distribution and nucleotide diversity of these elements appears to be the main distinction between different fungal taxa, making them promising candidates for diagnostic purposes. Fungi of the genus *Fusarium* display a high variation in MGE content, from MGE-poor (*Fusarium oxysporum* and *Fusarium fujikuroi* species complex) to MGE-rich mitogenomes found in the important cereal pathogens *F. culmorum* and *F. graminearum* sensu stricto. In this study, we investigated the MGE variation in these latter two species by mitogenome analysis of geographically diverse strains. In addition, a smaller set of *F. cerealis* and *F. pseudograminearum* strains was included for comparison. Forty-seven introns harboring from 0 to 3 endonucleases (HEGs) were identified in the standard set of mitochondrial protein-coding genes. Most of them belonged to the group I intron family and harbored either LAGLIDADG or GIY-YIG HEGs. Among a total of 53 HEGs, 27 were shared by all fungal strains. Most of the optional HEGs were irregularly distributed among fungal strains/species indicating ancestral mosaicism in MGEs. However, among optional MGEs, one exhibited species-specific conservation in *F. culmorum.* While in *F. graminearum* s.s. MGE patterns in *cox3* and in the intergenic spacer between *cox2* and *nad4L* may facilitate the identification of this species. Thus, our results demonstrate distinctive traits of mitogenomes for diagnostic purposes of Fusaria.

## Introduction

Most fungi contain mitochondria, organelles playing a key role in the generation of metabolic energy. Besides, fungal mitochondria have been shown to contribute to diverse cellular and organismal functions including senescence, quiescence, biofilm regulation and hyphal growth (Chatre and Ricchetti, 2014; Calderone et al., 2015; Bartelli et al., 2018; Sandor et al., 2018). They may also be involved in antifungal drug resistance, as well as in fungal virulence and pathogenicity (Shingu-Vazquez and Traven, 2011; Sandor et al., 2018). It is therefore not surprising that mitochondrial structure and function, as well as mitogenomes of fungi, have been studied intensively since the 2000s (Hausner, 2003; Chatre and Ricchetti, 2014).

Mitogenomes are expected to provide new insights for understanding the phylogenetic relationships and evolutionary biology of fungi (Aguileta et al., 2014; Lin et al., 2015; Franco et al., 2017). The reason for this is that fungal mitogenomes are highly divergent among even closely related lineages (Yin et al., 2012; Pogoda et al., 2019). This fact opens entirely new perspectives in diagnostics of fungi. The concept of applying mitogenomes for the identification of species mainly derives from their higher DNA copy number compared with nuclear DNA, and hence higher recovery and amplification success (Santamaria et al., 2009). However, for many fungal lineages, the characterization of mitochondrial DNA (mtDNA) has been largely limited by a low number of mitochondrial sequences in the GenBank database.

Fungal mitogenomes are double-stranded DNA molecules with relatively simple genetic structures often containing a set of 14 protein-coding genes, two rRNA coding genes and a large group of tRNA coding genes. The set of 14 core-genes encoding proteins is involved in the respiratory chain: the apocytochrome b (*cob*), 3 subunits of the cytochrome c oxidase (*cox* genes), 7 subunits of the NADH dehydrogenase (*nad* genes) and 3 components of the ATP synthase (*atp* genes). The two conserved rRNA genes encode the small (rns) and large (rnl) ribosomal RNA (rRNA) subunits (Bullerwell and Lang, 2005). In addition, fungal mitogenomes harbor a variable number of mobile genetic elements (MGEs) such as introns and associated homing endonucleases (HEGs), which have invaded the mitogenomes throughout the evolution (Basse, 2010; Joardar et al., 2012; Pogoda et al., 2019).

Surveys of intron distribution among different filamentous fungi have pinpointed a surprisingly high variation in MGE content, from the single-intron mitogenome of *Fusarium proliferatum* (Brankovics et al., 2017) to MGE-rich mitogenome of *Rhizoctonia solani* containing several dozens of MGEs (Losada et al., 2014). Most of the MGE insertion sites are highly conserved (Hausner, 2003) and occur in mitochondrial protein-coding genes, but certain genes can display remarkably different MGE densities (Hausner, 2003; Yin et al., 2012; Aguileta et al., 2014; Franco et al., 2017). In addition, the same MGEs can be irregularly distributed in evolutionarily distant species and mosaicism in MGE patterns can be found between different populations or even strains of the same species driving large genome size differences among them (Yin et al., 2012; Kolesnikova et al., 2019).

The widespread distribution of MGEs in fungi can be explained by an intron-rich progenitor of major eukaryotic lineages from which extensive and lineage-dependent intron loss has occurred. The most frequently assumed mechanism of intron loss considers the replacement of intron-containing genes with their intronless versions through homologous recombination between intronless cDNA and the corresponding genomic DNA (Hausner, 2003; Yin et al., 2012; Pogoda et al., 2019). In addition to intron/HEG loss, the infective nature of HEGs, which can propagate horizontally between different lineages is often suggested to drive the observed variation in MGE content. The mobility of HEGs is primarily explained by their transfer and site-specific integration, which usually involves three steps: recognition of an intronless allele, cleaving, and insertion of the HEG (Haugen et al., 2005; Yin et al., 2012). In addition, HEGs themselves may propagate over a variety of lineages independently from their host intron, resembling free-standing HEGs, which frequently occur in genomes of phages (Edgell, 2009; Franco et al., 2017). This type of mobility, although not frequently documented, drives variation in size and nucleotide content of introns (Wu and Hao, 2014).

Several recent studies addressed the comprehensive analyses of mitogenomes in important plant pathogenic *Fusarium* species (Al-Reedy et al., 2012; Fourie et al., 2013; Brankovics et al., 2017, 2018). Mitogenomes of Fusaria pose a typical set of mitochondrial genes with identical gene order. A unique feature apparently common in all *Fusarium* species is the presence of a large open reading frame with unknown function (LV-uORF) firstly described in mitogenomes of *F. graminearum*, *F. verticillioides* and *F. solani* (Al-Reedy et al., 2012). *Fusarium* mitogenomes have been shown to vary considerably in size. So far, *Fusarium pseudograminearum* contains the largest mitogenome of 110,526 bp long (described in this study), while the smallest proved only 30,629 bp long in *F. oxysporum* (Pantou et al., 2008). Such large size differences are caused primary by the irregular distribution of MGEs (Al-Reedy et al., 2012; Fourie et al., 2013; Brankovics et al., 2018). In *F. graminearum* sensu stricto (s.s.), MGEs comprise more than half of the mitogenome. Diversity in MGE content contributed to remarkable variation among geographically diverse strains which raised the question of the feasibility of using this type of variation in mitochondrial population genetic studies of *Fusarium* field populations (Brankovics et al., 2018).

*Fusarium graminearum* s.s. and *F. culmorum* are important and closely related plant pathogens exhibiting a mainly necrotrophic lifestyle after a short biotrophic infection stage and can be described as generalists based on their broad host ranges. Fusarium Head Blight (FHB) and Fusarium Crown Rot (FCR) are among the most important diseases of cereals caused by these pathogens. Both have led to major economic losses for the cereal-based feed and food supply chains. Besides these two species, other closely related fungi such as *F. cerealis* and *F. pseudograminearum* can be involved in cereal diseases (Leslie and Summerell, 2008).

All four pathogens exhibit a similar toxigenic potential by the production of type B trichothecenes and zearalenone (ZEA). These compounds are nowadays among the most frequently detected mycotoxins in different wheat- and barley-growing regions of the world (Desjardins, 2006). Trichothecenes have been found to induce mycotoxicoses in humans and animals and play a role in the virulence of fungal strains (Desjardins, 2006). ZEA is a non-steroidal estrogen that may cause female reproductive changes due to its strong estrogenic activity (Hueza et al., 2014).

However, despite the aforementioned similarities, all four *Fusarium* species appear to exhibit considerable variation in distribution and frequency in agroecosystems. The most noticeable, despite their frequent co−existence, is the dramatic prevalence of *F. graminearum* s.s. in many cropping regions of the world (van der Lee et al., 2015). The emergence of *F. graminearum* s.s. resulted in the continuous displacement of other FHB causing *Fusarium* species. The best example illustrating this displacement is the gradual decrease of *F. culmorum*, which has been traditionally reported as the primary cause of FHB in Northern, Central and Western Europe (Waalwijk et al., 2003; Scherm et al., 2013; Bilska et al., 2018a). *F. cerealis* causes root rots and seedling blights, but can also be found on corn cobs and as emerging, mycotoxigenic FHB pathogen in temperate regions (Amarasinghe et al., 2015). While the predominance of *F. pseudograminearum* has been linked to the rather warm and dry environmental conditions (Kazan and Gardiner, 2018).

Even when using a software package designed for patch characterization in images, *Fusarium cerealis* could neither be distinguished unambiguously from *F. sambucinum* nor from *F. graminearum s. s.* based on their morphological spore shape parameters (Dubos et al., 2012). All of them produce similar banana-shaped macroconidia and chlamydospores. *F. graminearum* s.s. is morphologically identical to *F. pseudograminearum*, while *F. culmorum* can easily be confused with *F. cerealis* (Leslie and Summerell, 2008). More reliable identification of these four species could be achieved by using molecular markers, especially with methods employing species-specific primers in either end point (conventional PCR) or qPCR (Waalwijk et al., 2004; Nicolaisen et al., 2009). A more laborious method of species discrimination is based on multi-locus sequencing (MLST) (Geiser et al., 2004; Wang et al., 2011). However, the major disadvantage of nuclear markers is their reduced detection limits mostly due to the targeted nuclear single-copy genes (Kulik et al., 2015; Bilska et al., 2018b). Amongst the most promising solutions is targeting repeated genomic sequences such as mitochondrial DNA. However, the low number of available mtDNA sequences from Fusaria so far limited the evaluation of mitochondrial DNA as a diagnostic marker.

Today, the growing body of mitogenome data suggests that variation in MGE number can follow the divergence of different fungal taxa (Brankovics et al., 2017; Liang et al., 2017; Fourie et al., 2018), offering great perspectives for future diagnostic purposes. Brankovics et al. (2017) showed that mosaicism in intron patterns can support the separation of three phylogenetic species in *F. oxysporum*. Fourie et al. (2018) revealed significant differences in intron patterns among species from the *Fusarium fujikuroi* species complex (FFSC). Beyond *Fusarium*, recent studies on *Colletotrichum* mitogenomes indicated that intron content variation could improve cryptic species delimitation within this genus (Liang et al., 2017).

This study demonstrates that the variable landscape of MGEs is the most prominent type of variation among mitogenomes of closely related *F. graminearum* s.s. and *F. culmorum*. The major objective of this study was to discover if the pattern of MGE diversity might facilitate the delimitation of these species.

Therefore, we sequenced, assembled and annotated the mitogenomes from geographically diverse strains. Mitogenomes of the studied Fusaria were MGE-rich and most of them were present in all studied strains. Protein homology searches in GenBank database revealed a number of HEG orthologs shared with other fungal species with considerable variation in protein identity scores. Among the total of 53 identified HEGs, one showed species-specific conservation in *F. culmorum*. *F. graminearum* s.s. did not harbor unique MGEs throughout the mitogenome, however, we found that MGE patterns in *cox3* and in the intergenic spacer between *cox2* and *nad4L* that can serve as potential markers for the identification of this species.

## Materials and Methods

### Fungal Strains

Supplementary File 1 provides characteristics of all fungal strains used in this study. A more detailed description of the strains can be found in the ToxGen database (Kulik et al., 2017). The mitogenomes of 78 strains of *F. graminearum* s.s. and 33 strains of *F. culmorum* were included into the analyses. In addition, 8 strains of *F. cerealis* and 4 strains of *F. pseudograminearum* were included for comparison. Moreover, 23 complete mitogenomes of *F. graminearum* s.s. previously described by Brankovics et al. (2018) and one mitogenome of *F. culmorum* (Kulik et al., 2016a) were also included.

### DNA Extraction and Sequencing

For DNA extraction, fungal strains were incubated on Petri plates (Ø 80 mm) with PDA medium at 24°C for 6 days. DNA from fungal strains was extracted from 0.1 g of mycelium with the use of the Quick-DNA Plant/Seed Miniprep Kit (Zymo Research, Irvine, CA, United States) according to the manufacturer’s protocol.

Whole-genome sequencing was performed at Macrogen (Seoul, South Korea). Genome libraries were constructed using a TruSeq DNA PCR-free library preparation kit (Illumina, San Diego, CA, United States). An Illumina HiSeq X Ten platform was used to sequence the genomes using a paired-end read length of 2 × 150 bp with an insert size of 350 bp.

### Assembly and Annotation of Mitogenomes

NOVOPlasty 2.7.2 (Dierckxsens et al., 2017) was used for *de novo* assembly of fungal mitogenomes from whole-genome data. HEGs were identified using NCBI’s ORF Finder^1^, Blast+ (v. 2.9.0) (Johnson et al., 2008) and Geneious Prime software (Biomatters Ltd., New Zealand). Annotations were performed using MFannot^2^, InterPro (Mitchell et al., 2015), CD-Search (Marchler-Bauer and Bryant, 2004) and Geneious Prime software (Biomatters Ltd., New Zealand). Annotation of tRNA genes was improved using tRNAscan-SE (Pavesi et al., 1994).

Complete mitogenomes have been deposited in the NCBI database under the GenBank accession numbers MT036635-757 (Supplementary File 1).

### Alignment

Mitogenome comparison included a total of 147 complete mitogenomes. Multiple sequence alignments of the complete mitogenomes were performed with progressive Mauve (Darling et al., 2010) implemented in the Geneious Prime software (Biomatters Ltd., New Zealand) to examine the distribution of MGEs in fungal mitogenomes.

### BlastP Analyses

To reveal the distribution and the diversity of HEGs, blastP searches were performed using the BLAST program available at the National Center for Biotechnology Information web page https://www.ncbi.nlm.nih.gov/. Hits were retained only if they had an *e*-value cut off lower than 0.001 and which covered at least 70% of the query sequence with >60% identity.

### Phylogenetic Analysis

HEGs showing higher protein similarity to sequences from distantly related species than to those found in other Fusaria were subjected for phylogenetic analysis. Protein sequences were aligned using the Muscle algorithm implanted in the software package Geneious Prime (Biomatters Ltd., New Zealand). An optimal evolutionary model was estimated using MEGA X (Kumar et al., 2018). The trees were constructed using the Maximum Likelihood method as implemented in Mega X with JTT + G + F model and 2000 bootstrap replicates.

## Results

### General Features of Fungal Mitogenomes

The mitochondrial genomes of all strains contained a conserved set of genes in the same order and orientation; a set of 14 protein-coding genes, 2 rRNA genes (*rns* and *rnl*) and 28 tRNA genes. The four *Fusarium* species displayed a similar GC content of 31.6–32.0% within their mitogenomes; clearly in the middle limit of the GC content range for fungal mitogenomes (8.4–52.7%, estimated from sequence data deposited in the GenBank database of the NCBI by April 2019).

Mitogenome sizes ranged from of 92.816 bp (*F. cerealis* strain 64722) to 110.526 bp for (*F. pseudograminearum* strain CBS 109956) (Supplementary File 1). These sizes of mitogenomes are well in between the current size limits of the fungal mitogenomes ranging from 12 kb to over 235 kb (based on GenBank database by April 2019). To date, the mitogenome of *F. pseudograminearum* remains the largest among fungi of the genus *Fusarium*.

Besides genes with functional predictions, *Fusarium* mitogenomes contained a large open reading frame with unknown function (LV-uORF). This ORF is located in the intergenic spacer between *rnl* and the *nad2* gene (Al-Reedy et al., 2012). The GC content of the LV-uORF is higher than the usual set of mitochondrial genes suggesting its horizontal acquisition by a common ancestor of *Fusarium* species (Al-Reedy et al., 2012).

**FIGURE 1:**
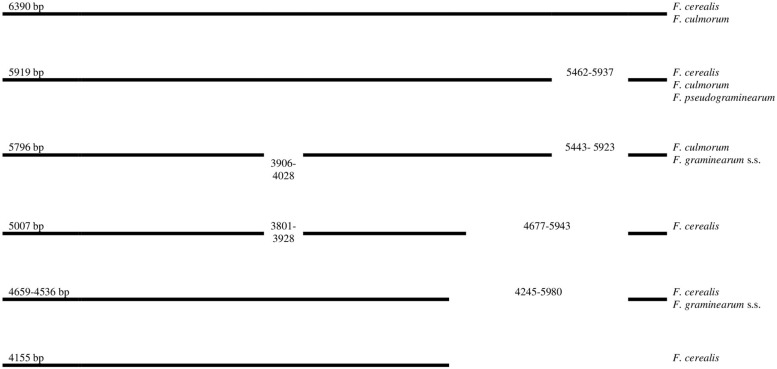
Schematic position of indels in LV-uORF in four Fusarium species. Position of indels (bp) are referred to *F. cerealis* (GenBank accession number MT036645, CBS 110268 strain).

Our results showed a highly conserved distribution of LV-uORF in four studied species (Supplementary File 1). We found that LV-uORF is present in all examined strains in the same position and orientation. However, we found considerable variation within LV-uORF mainly due to multiple indel mutations altering its length, which ranged from 4.155 to 6.390 bp (Figure 1). The apparently conserved placement of indels within LV-uORF and their irregular distribution among strains and species suggests that most of these events occurred prior to the divergence of the *Fusarium* species.

### Mobile Genetic Elements

A total of 47 introns has been identified in the basic set of mitochondrial protein-coding genes of the studied strains (Supplementary Files 1, 2a–6a). All introns belonged to the group I intron family with two exceptions: the first one was *iIIcob* intron, which was classified to the group II introns and encodes a protein of the reverse transcriptase family (Supplementary File 2a). Notably, the distribution of this intron within studied Fusaria seems to be very limited. We detected its presence in only 2 *F. graminearum* s.s. strains (16-462-z and INRA-156). The second exception was the *i4cox2* intron, which lacked distinguishable features for classification (Supplementary File 3a).

Most introns harbored HEGs, either of the LAGLIDADG or GIY-YIG type, which are determined based on the differences in conserved amino acid motifs. Among 53 distinct HEGs, 36 were assigned as LAGLIDADG endonucleases being present in 9 out of 11 intron-rich genes. GIY-YIG endonucleases appear to be less commonly occurring HEGs in mitogenomes of Fusaria. We identified 15 distinct GIY-YIG endonucleases in only six out of eleven protein-coding genes. However, two HEGs [*i4cob* HEG (112 aa) and *i6cob* HEG (472 aa)] could not be precisely predicted through similarity searches with blastP, presumably due to loss of conserved amino acid motifs that reflect these functional differences. We found that orthologs of *i4cob* HEG present in GenBank are much larger in the other species; e.g., in *Podospora comata* this HEG is 314 aa in length (YP_009550007.1), while in *Neurospora crassa* – 477 aa (YP_009126715.1), which suggests deletion events during the divergence of Fusaria. The second *i6cob* HEG showed the highest identity (73.8 – 75.5%) to unclassified mitochondrial proteins (ATI20208.1, ATI20295.1, ATI20458.1) found in *Juglanconis* sp. (q-cover = 99-100%, *E* value = 0).

More than half of the introns (*n* = 26) identified in Fusaria were located in 3 subunits of the cytochrome c oxidase (*cox* genes): 17 in *cox1*, 5 in *cox2*, and 4 in *cox3* (Supplementary Files 2a–6a). Positions of MGEs were highly conserved among different strains, however, with one exception. We found that two different introns *icox1a* and *icox1b* can share the same location in *cox1* (Supplementary File 4a). Intron *icox1a* harbored a LAGLIDADG motif and was present in *F. cerealis*, while *icox1b* contained a GIY-YIG motif and was found in all strains of *F. culmorum*, *F. graminearum* s.s. and *F. pseudograminearum*. Further analyses of a larger collection of strains could confirm whether the LAGLIDAD HEG found in *icox1a* is fixed in *F. cerealis.*

**FIGURE 2:**
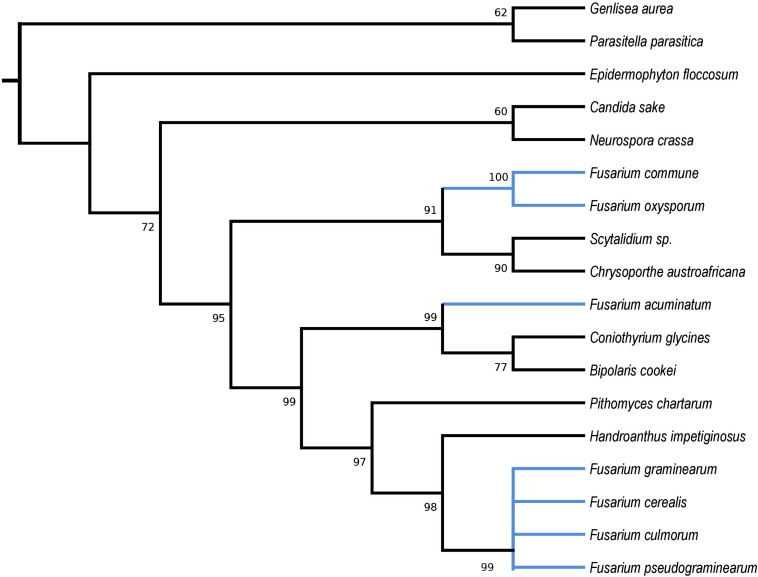
Maximum Likelihood tree inferred from *i1nad5* LAGLIDADG HEG. GenBank accession numbers are given in brackets *Bipolaris cookie* (YP_009445560.1), *Candida sake* (YP_008475145.1), *Chrysoporthe austroafricana* (YP_009262028.1), *Coniothyrium glycines* (YP_009543504.1), *Epidermophyton floccosum* (YP_313631.1), *Fusarium acuminatum* (CDL73463.1), *Fusarium cerealis* (YP_009741100.1), *Fusarium commune* (YP_009437834.1), *Fusarium culmorum* (CDL73494.1), *Fusarium graminearum* (YP_001249315.1), *Fusarium oxysporum* (ATG24573.1), *Fusarium pseudograminearum* (CDL72485.1), *Genlisea aurea* (EPS67323.1), *Handroanthus impetiginosus* (PIM96989.1), *Neurospora crassa* (YP_009126712.1), *Parasitella parasitica* (YP_009059710.1), *Pithomyces chartarum* (YP_009415186.1), and *Scytalidium* sp. (QDG01253.1).

**FIGURE 3:**
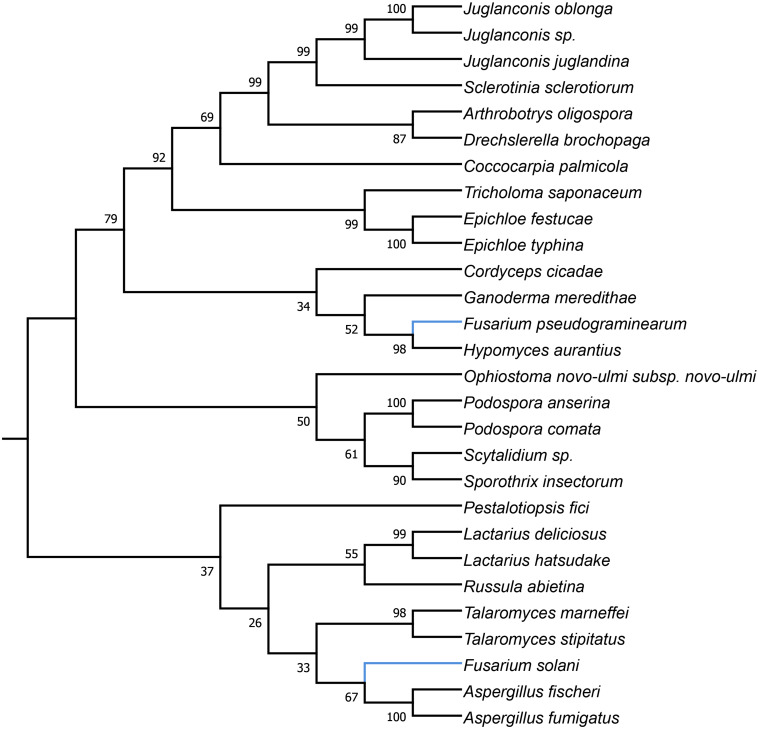
Maximum Likelihood tree inferred from *i3cox1* LAGLIDADG HEG. GenBank accession numbers are given in brackets *Arthrobotrys oligospora* (QID02895.1), *Aspergillus fischeri* (AFD95944.1), *Aspergillus fumigatus* (YP_005353060.1), *Coccocarpia palmicola* (YP_009355407.1), *Cordyceps cicadae* (YP_009577892.1), *Drechslerella brochopaga* (QCW06877.1), *Drechslerella brochopaga* (YP_009568474.1), *Epichloe festucae* (YP_009327861.1), *Epichloe typhina* (YP_009327804.1), *Fusarium pseudograminearum* (QID41639.1), *Fusarium solani* (YP_005088111.1), *Ganoderma meredithae* (YP_009129958.1), *Hypomyces aurantius* (YP_009254043.1), *Juglanconis juglandina* (ATI20474.1), *Juglanconis oblonga* (ATI20215.1), *Juglanconis* sp. (ATI20300.1), *Lactarius deliciosus* (YP_009498182.1), *Lactarius hatsudake* (YP_009504192.1), *Ophiostoma novo-ulmi* (AVD96803.1), *Pestalotiopsis fici* (YP_009317077.1), *Podospora anserina* (NP_074927.1), *Podospora comata* (YP_009550011.1), *Russula abietina* (YP_009487192.1), *Sclerotinia sclerotiorum* (YP_009389070.1), *Scytalidium* sp. (QDG01205.1), *Sporothrix insectorum* (QGX43780.1), *Talaromyces marneffei* (NP_943724.1), *Talaromyces stipitatus* (AFD95918.1), and *Tricholoma saponaceum* (QIC20272.1).

**FIGURE 4:**
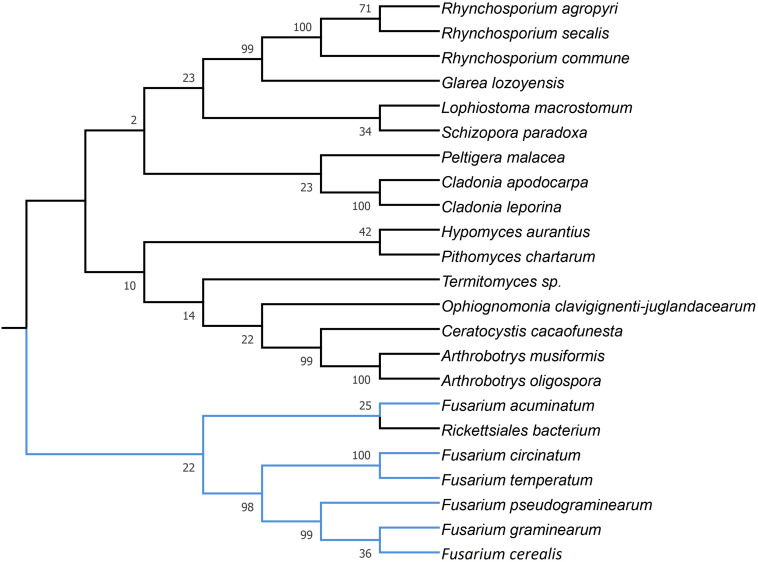
Maximum Likelihood tree inferred from *i13cox1* LAGLIDADG HEG. GenBank accession numbers are given in brackets *Arthrobotrys musiformis* (YP_009574367.1), *Arthrobotrys oligospora* (QID02793.1), *Ceratocystis cacaofunesta* (YP_007507075.1), *Ceratocystis cacaofunesta* (YP_007507075.1), *Cladonia apodocarpa* (YP_009514117.1), *Cladonia leporina* (YP_009526663.1), *Fusarium acuminatum* (CDL73453.1), *Fusarium circinatum* (YP_008757706.1), *Fusarium cerealis* (YP_009741121.1), *Fusarium graminearum* (YP_001249335.1), *Fusarium pseudograminearum* (CDL72517.1), *Fusarium temperatum* (AKM98021.1), *Glarea lozoyensis* (AGN74496.1), *Glarea lozoyensis* (YP_009306750.1), *Hypomyces aurantius* (YP_009254047.1), *Lophiostoma macrostomum* (KAF2647136.1), *Ophiognomonia clavigignenti-juglandacearum* (ATI20563.1), *Peltigera malacea* (YP_005351183.1), *Pithomyces chartarum* (YP_009415166.1), *Rhynchosporium agropyri* (YP_008965308.1), *Rhynchosporium commune* (YP_008965356.1), *Rhynchosporium secalis* (YP_008965429.1), *Rickettsiales bacterium* (RYE15942.1), *Schizopora paradoxa* (YP_009690425.1), and *Termitomyces* sp. (AYE93211.1).

**FIGURE 5:**
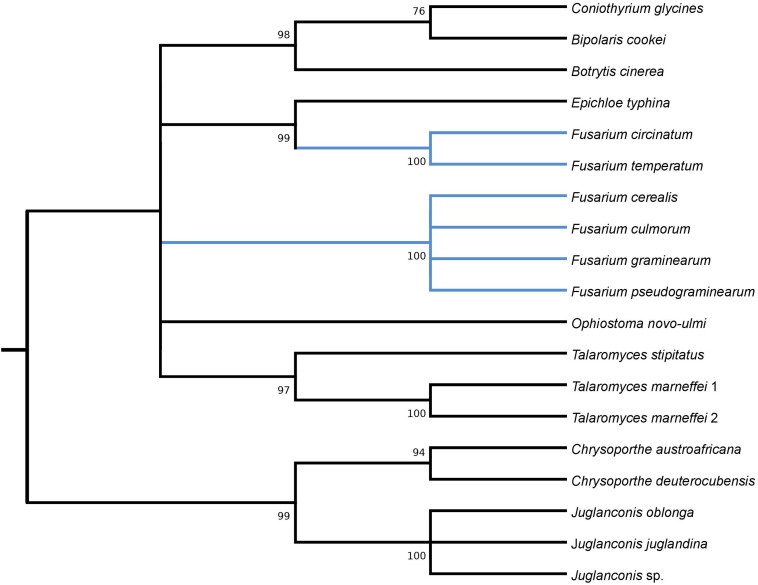
Maximum Likelihood tree inferred from *i14cox1* GIY-YIG HEG. GenBank accession numbers are given in brackets *Bipolaris cookei* (YP_009445523.1), *Botrytis cinerea* (AGN49010.1), *Chrysoporthe austroafricana* (YP_009262000.1), *Chrysoporthe deuterocubensis* (YP_009262086.1), *Coniothyrium glycines* (YP_009543497.1), *Epichloe typhina* (YP_009327808.1), *Fusarium cerealis* (YP_009741122.1), *Fusarium culmorum* (CDL73523.1), *Fusarium graminearum* (YP_001249336.1), *Fusarium pseudograminearum* (QID41650.1), *Fusarium circinatum* (YP_008757707.1), *Fusarium temperatum* (AKM98023.1), *Juglanconis juglandina* (ATI20489.1), *Juglanconis oblonga* (ATI20223.1), *Juglanconis* sp. (ATI20312.1), *Ophiostoma novo-ulmi* (AVD96809.1), *Talaromyces marneffei* 1 (AFD95988.1), *Talaromyces marneffei* 2 (NP_943730.1), and *Talaromyces stipitatus* (AFD95924.1).

HEGs drive mobility of MGEs, which can move across species, genera or even kingdoms (Koufopanou et al., 2002; Wu and Hao, 2014). We performed comprehensive blastP searches against GenBank to gather information on their distribution and the diversity among species. BlastP analyses revealed that most of the identified HEGs can be found in other *Fusarium* species as well as in species out of the genus *Fusarium*, however, with considerable variation in protein identity scores (Supplementary Files 2b–6b). Notably, for most HEGs (49 out of 53), protein identity was greater within than outside the genus *Fusarium*, which indicates their ancestral acquisition in the genus *Fusarium*. However, 4 HEGs showed higher similarity to sequences from distantly related species than to those found in the other Fusaria, suggesting their more recent acquisitions from distantly related donors. These include LAGLIDADG HEGs found in introns: *i1nad5, i3cox1*, *i13cox1* and single *i14cox1* GIY-YIG HEG. To test their horizontal transfer, we attempted to construct maximum-likelihood (ML) amino acid trees using GenBank records covering at least 85% of the query protein sequence (Figures 2–5).

Phylogenetic analyses provided strong evidence for horizontal gene transfer in the case of *i1nad5* LAGLIDADG HEG (Figure 2). On this particular tree, the LAGLIDADGs from different Fusaria are scattered in three different well-supported clades together with HEGs harbored by more distantly fungi such as *Bipolaris cookei*, *Chrysoporthe austroafricana*, *Coniothyrium glycines*, *Pithomyces chartarum*, and *Scytalidium* sp. Maximum Likelihood analyses also provided strong evidence for different evolutionary histories of two *i3cox1* LAGLIDADG HEGs present in *F. pseudograminearum* and in *F. solani*. Phylogenetic trees depict that both HEGs cluster into a different clades (Figure 3). The HEG found in *F. pseudograminearum* forms a single cluster with *Hypomyces aurantius*, while the *F. solani* HEG shows its closest relationship to orthologs found in *Aspergillus* spp. However, phylogenetic analysis failed to support a different phylogenetic history of *i13cox1* HEG in Fusaria. Many nodes on the tree had low bootstrap support (Figure 4). Supplementary File 4b depicts distribution of this HEG among fungal lineages. It displays high conservation among *F. cerealis*, *F. culmorum*, *F. graminearum* s.s., *F. pseudograminearum, F. circinatum* and *F. temperatum* (Identity = 90–100%, q-cover = 89–100%, *E*-value = 0), but only 61% identity to HEG found in *F. acuminatum.* BlastP analyses indicated its higher similarity (70–76%) to orthologs present in more distantly species such as *Hypomyces aurantius*, *Ceratocystis cacaofunesta*, *Peltigera malacea*, *Arthrobotrys* spp., *Glarea lozoyensis*, *Cladonia* spp., *Termitomyces* sp., *Rhynchosporium* spp. and *Pithomyces chartarum*. We hypothesize that the incorrect conclusion from this phylogenetic analysis may be likely due to high divergence within groups and a reduced number of taxa in the data set. The evidence for horizontal transfer of HEG between different fungal species is also indicated in the case of the *i14cox1* GIY-YIG HEG tree by demonstrating a closer relationship of *F. circinatum*/*F. temperatum* to *Epichloe typhina* than to the remaining Fusaria (Figure 5).

### The Majority of MGEs Show Highly Conserved Distribution in *Fusarium* Strains/Species

Twenty-six introns and 27 HEGs were shared by all fungal strains. The majority of them showed high sequence conservation. Eleven from these introns showed very high similarity with no size variation and pairwise identity ranging from 99.9 to 100%. 12 introns displayed some size variation due to small, strain-dependent indels mostly below 100 bp in size. When excluding indel variation, their nucleotide diversity was very low, with pairwise identity above 99%. The last three introns: the *i11cox1*, the *i4cob* intron, and the *i1atp6* intron showed huge size variation (Supplementary Files 2a, 4a, 5a). Among these conserved introns, all contained two LAGLIDADGs among which the first was conserved (invariably present in all studied strains), while the second one (optional) was irregularly distributed among the strains, presumably due to independent of introns spread of HEGs. However, the distribution of optional HEGs across multiple strains was low. An optional *i11bcox1* LAGLIDADG HEG (318 aa) was found in only one fungal mitogenome (CBS 119173). Only four fungal strains (all *F. pseudograminearum*) harbored optional LAGLIDADG HEG (387 aa) in the *atp6* gene. The occurrence of the third optional *i4cob* LAGLIDADG HEG (252 aa) was more frequent, but still low, being found in 14% of strains. We also found that the unexceptionally short (112 aa) *i4cob* HEG displays optional distribution among fungal strains and was more frequent than the other optional HEGs being found in 27% of strains (Supplementary Files 1, 2a).

### HEG Content Variation Between in *F. culmorum* and in *F. graminearum* s.s.

Among 53 HEGs identified in introns, 26 were optional (irregularly distributed among all strains). We analyzed their distribution in the context of species-specific occurrence in *F. culmorum* and in *F. graminearum* s.s. Mitogenomes of *F. cerealis* and *F. pseudograminearum* were not analyzed in the context of species-specific distribution of MGEs due to low number of representative strains. We found that among these optional HEGs, one located in *i3acox2* intron was fixed in *F. culmorum* (Supplementary Files 3a,b). We also found another *i4cox3* LAGLIDADG HEG in *F. culmorum* which was absent in four closely related Fusaria, but BlastP searches indicated its presence in other, more distantly *F. solani* (Supplementary Files 3a,b), however, with 23% difference in protein identity scores. Noteworthy, its nucleotide sequence appears to be highly conserved in *F. culmorum* fulfilling criterion of diagnostic marker, which should display enough conservation to achieve less variability within species than between species (Raja et al., 2017).

The absence of unique MGEs in *F. graminearum* s.s. prompted us to search for unique MGE patterns in *F. graminearum* s.s. Among protein-coding genes, we found that intron content variation in *cox3* exhibits unique mosaicism in *F. graminearum* s.s. *Cox3* contains four introns (Supplementary File 3a), among which two: *i1cox3* and *i2cox3* are present in all species. The third *i3cox3* intron is present in *F. cerealis* and *F. pseudograminearum*, while the fourth the *i4cox3* is uniquely present in *F. culmorum*. Thus, the lack of these last two introns in *F. graminearum* s.s. determines a unique MGE pattern in *F. graminearum* s.s.

Besides HEGs found in protein-coding genes, the mitogenomes of three Fusaria (*F. cerealis, F. culmorum*, and *F. pseudograminearum*) contained an approximately 1895 bp insertion between *cox2* and *nad4L*, which is absent in *F. graminearum* s.s. This insertion harbors two ORFs: ORF 313 (942 bp) and ORF 132 (399 bp), both encoding LAGLIDADG HEGs. From a diagnostic point of view, the absence of these two HEGs in *F. graminearum* s.s. also appears to be promising diagnostic features separating *F. graminearum* s.s. from the remaining three species.

## Discussion

Highly sensitive and precise detection and quantification of fungi are critical to support diagnosis for disease surveillance. Therefore, this research field has been revolutionized by the advent of molecular markers (Tsui et al., 2011). However, the detection of fungi is still a challenge, because fungal biomass is often present at very low and uneven concentrations in environmental or clinical samples. For that reason, the use of DNA extraction methods facilitating efficient recovery of fungal DNA is highly recommended, however, with even highly efficient extraction techniques, low amounts of fungal DNA are often extracted in the presence of large amounts of background DNA, hampering largely the sensitivity of the assays (Wickes and Wiederhold, 2018).

The use of assays targeting repeated genomic sequences such as internal transcribed spacer (ITS) of the rRNA region (official fungal barcode) provides one of the best solutions to this problem (Yang et al., 2018). Unfortunately, ITS fails to delineate most agriculturally important *Fusarium* species (Schoch et al., 2012). Consequently, for most Fusaria, diagnostic markers enabling their species-specific identification, have been developed based on single-copy nuclear loci (Nicolaisen et al., 2009; O’Donnell et al., 2015), which can limit successful detection of low amounts of fungal biomass (Kulik et al., 2015; Bilska et al., 2018b).

Targeting mitochondrial DNA could be a viable solution due to its high copy number in fungal cells (Krimitzas et al., 2013). The use of mtDNA for barcoding of fungi has been studied more than a decade ago (Seifert et al., 2007). In one of the first in-depth studies, it was demonstrated that the standard animal barcode *cox1* could be highly effective in the resolution of *Penicillium* species. However, *cox1* showed significant diagnostic limitations for *Fusarium* fungi (Gilmore et al., 2009) mostly due to the presence of paralogous copies of *cox1* in studied strains and low species resolution. Besides, one of the major shortcomings of mitogenome-based studies in fungi is the difficulty in obtaining amplicons for further sequencing, mainly due to large indel polymorphism associated with irregular distribution of MGEs. Further study of Santamaria et al. (2009) showed that most mitochondrial genes of *Ascomycota* fungi contain MGEs, which largely limits their potential use for barcoding purposes.

Emerging next-generation sequencing provides a new opportunity for the rapid characterization of fungal mitogenomes. Mitogenomes can be assembled from the whole genome reads via either *de novo* assemblies or mapping to a reference genome (Tenaillon et al., 2011) providing an unbiased look into the patterns of MGE diversity. It is worth noting that exploration of this type of diversity on species or population level was previously not possible due to low numbers of complete mitogenomes. In one of the first large-scale studies on budding yeast, *Saccharomyces cerevisiae*, Wolters et al. (2015) revealed that the distribution of certain MGEs follows species divisions and even exhibits population-specific profiles. Further, but still, very limited studies on plant pathogenic fungi appeared to support this evidence. Despite the observed strain-dependent variation in MGE content, some of these elements appear to show species-specific occurrence in *F. oxysporum* (Brankovics et al., 2017), *Fusarium fujikuroi* species complex (FFSC) (Fourie et al., 2018) or more distantly related *Colletotrichum* spp. (Liang et al., 2017). In this study, we identified a single *i3acox2* HEG displaying species-specific conservation in *F. culmorum* (Supplementary Files 3a,b). A previous study by Bilska et al. (2018b) demonstrated that targeting of this HEG through qPCR technology offers highly specific and sensitive quantification of *F. culmorum.*

We found that positions of MGEs were highly conserved among different strains. More than half of introns (*n* = 26) were found in *cox* genes (Supplementary Files 2a–6a), which is in line with accumulating evidence from various fungal lineages confirming remarkably high density in MGE content in especially *cox1* (Sandor et al., 2018; Zubaer et al., 2018; Pogoda et al., 2019). This, however, is notably in contrast to recent findings which showed that other Fusaria display rather low intron density within their *cox* genes, which can be even intronless in species from *F. oxysporum* (Brankovics et al., 2017) and *Fusarium fujikuroi* species complex (FFSC) (Fourie et al., 2018). However, it is worth to note that mitogenomes of the strains analyzed in our study are MGE−rich in comparison with previously studied Fusaria (Brankovics et al., 2017; Fourie et al., 2018). Notably, there were large differences in frequency distribution of MGEs. The vast majority of them occur at relatively higher frequencies compared to others, such as the *iIIcob* intron or the *i11bcox1* LAGLIDADG HEG (318 aa), raising the question on their role in gene regulation and their maintenance through selection (Guha et al., 2018; Zubaer et al., 2018).

We found that among 53 HEGs identified in introns, 9 showed irregular distribution within *F. graminearum* s.s., and only 4 within *F. culmorum* (Supplementary Files 2a–6a). The increased variation in HEG content within *F. graminearum* s.s. may be explained by its recombining reproductive behavior (outcrossing) (Trail, 2009; Talas and McDonald, 2015), which is assumed to favor intron mobility (Hausner, 2003). Unlike in *F. graminearum* s.s., no evidence of sexual reproduction was found in both *F. culmorum* and *F. cerealis* (Leslie and Summerell, 2008; Scherm et al., 2013). Sexual recombination can occur in *F. pseudograminearum*; however, it was rarely observed under field conditions (Summerell et al., 2001). Previous population genomic analyses by Kelly and Ward (2018) (restricted to North American populations) showed that in *F. graminearum* s.s., gene content variation in the nuclear genome displays population-specific patterns of conservation. We attempted to find out if variation in the MGE content shows population-specific profiles. To achieve it, we investigated if the distribution of optional HEGs reflects the geographic origin of the strains. We found that such a link does not exist. The same HEG patterns could be found in strains originated from distant geographic locations as in strains isolated from the same and even relatively small geographic locations (e.g., Luxembourg), which is indicative of low mobility of ancestrally acquired MGEs shared by different populations of this phylogenetic species. Our findings are, however, in sharp contrast to previous survey on *Saccharomyces cerevisiae* showing that the MGEs can be distributed in a population-specific manner (Wolters et al., 2015).

Assuming low mobility of MGEs in Fusaria, it could be speculated that HEG content variation will not follow cryptic divergence of other phylogenetic species from the *Fusarium graminearum* species complex (FGSC). Until now, only one complete mitogenome of *F. gerlachii* (Kulik et al., 2016b) is available in the GenBank database. Indeed, analysis of its mitogenome did not reveal unique MGEs separating *F. gerlachii* from *F. graminearum* s.s. (Supplementary Files 2b–6b). Besides MGE content variation, we previously showed that SNP polymorphism found in conserved introns can facilitate design of species-specific markers in FGSC. A previously developed mitochondrial-based qPCR assay has been designed based on such a polymorphism for species-specific detection and quantification of *F. graminearum* s.s. (Kulik et al., 2015). The assay has been demonstrated not to interfere with other phylogenetic species from FGSC, which indicates that this type of polymorphism could be useful in the assessment of cryptic diversity in Fusaria. This issue will need to be fully addressed in our further studies by analysis of mitogenomes from all known cryptic species from FGSC.

Blast alignments are commonly employed to reveal different evolutionary histories of genes (Zhu et al., 2014). In this study, protein homology searches in GenBank database showed that most HEGs found in four *Fusarium* species are shared with other fungal species (Supplementary Files 2b–6b). This variation was very low when comparing HEG orthologs from closely related *F. cerealis*, *F. culmorum*, *F. gerlachii*, *F. graminearum* s.s. and *F. pseudograminearum*, and increased when including other Fusaria. In most cases variation in protein identity scores was highest for HEG orthologs from other fungal genera, which is indicative of an ancestral acquisition of HEGs in the genus *Fusarium*.

MGEs, when fixed in population, are susceptible to degeneration. To ensure their survival, these elements can spread to new populations or even horizontally across species (Koufopanou et al., 2002; Wu and Hao, 2014). HEGs are well-adapted for horizontal transmission by the maintenance of highly conserved recognition sequences and the conserved nucleotide sites most critical for homing (Koufopanou et al., 2002). However, in this study, we found, that among a total of 53 HEGs, only 4 showed hallmarks of horizontal mobility across different fungi (Figures 2–5). Our analyses also suggest two cross-kingdom horizontal gene transfer events (Figures 2, 4). The first one is indicated by a higher (66.91%) protein identity (q-cover = 88%, *E* value = 3e-115) of *i1nad5* LAGLIDADG HEG (Supplementary Files 6a,b) to the plant ortholog (PIM96989.1) found in *Handroanthus impetiginosus* than to the other fungal orthologs. The second event is indicated by higher (63.6%) identity of *i13cox1* LAGLIDADG HEG (Supplementary Files 4a,b) to ortholog found in *Rickettsiales bacterium* (RYE15942.1) (q-cover = 94%, *E* value = 9e-174) than to the other fungal derived orthologs. BlastP searches showed that both HEGs gave no significant BLAST hits in species outside fungi, which suggests a cross-kingdom jump from a fungal donors to the plants (PIM96989.1) and alphaproteobacteria (RYE15942.1).

Overall, our results presented in this study demonstrate that the MGE diversity in fungal mitogenomes may lead to the development of novel diagnostic markers offering improved detection of fungi. Diagnostic markers for pathogenic fungi need to be tested against the range of strains from different geographical origins to ensure their uniformity. The geographically diverse mitogenomes provide valuable resources for the marker design. However, in case of *F. cerealis* and *F. pseudograminearum* more strains are needed for robust conclusions. Today, mitochondrial-based qPCR assays are available for both *F. culmorum* and *F. graminearum* s.s. (Kulik et al., 2015; Bilska et al., 2018b). qPCR remains a valuable option due to the ability to quantify fungal DNA, especially from difficult food or environmental samples from which extraction of high-molecular-weight DNA is problematic. In addition, the increased sensitivity of mitochondrial-based markers may reduce the incidence of scoring errors, making interpretation qPCR results less error-prone. Besides qPCR, other promising techniques such as loop-mediated isothermal amplification (LAMP) may be also considered (Panek and Frac, 2019). Among a number of molecular methods for the identification of fungi, barcoding DNA provides the best potential for biodiversity monitoring (Begerow et al., 2010). However, it would require multiple group-specific primers for sequencing of MGEs-barcodes, which is nowadays time-consuming and cost-intensive. Whatever the method, the revealed strong evidence for species-specific polymorphism in fungal mitogenomes opens entirely new perspectives to fungal diagnostics, which can be of great benefit in the field of agriculture and food security when highly sensitive detection of fungi is needed.

## Conclusion

Mitogenomes of *F. culmorum* and *F. graminearum* s.s. are MGE-rich and harbor especially group I introns encoding LAGLIDADG and GIY-YIG HEGs. The majority of mitochondrial MGEs showed a highly conserved distribution in all studied strains. MGEs present in these species can be found in other Fusaria and in other fungal genera with considerable variation in protein identity scores. Intraspecies variation in MGE content is more evident in *F. graminearum* s.s. than in *F. culmorum* but is indicative of ancestral mosaicism of HEGs shared by different fungal populations. Among optional HEGs, one exhibits species-specific conservation in *F. culmorum. F. graminearum* s.s. does not harbor unique MGEs throughout the mitogenome, however, MGE patterns in *cox3* and in the intergenic spacer between *cox2* and *nad4L* may support identification of this species.

## Data Availability Statement

The datasets generated for this study can be found in the NCBI GenBank database. MT036635, MT036636, MT036637, MT036638, MT036639, MT036640, MT036641, MT036642, MT036643, MT036644, MT036645, MT036646, MT036647, MT036648, MT036649, MT036650, MT036651, MT036652, MT036653, MT036654, MT036655, MT036656, MT036657, MT036658, MT036659, MT036660, MT036661, MT036662, MT036663, MT036664, MT036665, MT036666, MT036667, MT036668, MT036669, MT036670, MT036671, MT036672, MT036673, MT036674, MT036675, MT036676, MT036677, MT036678, MT036679, MT036680, MT036681, MT036682, MT036683, MT036684, MT036685, MT036686, MT036687, MT036688, MT036689, MT036690, MT036691, MT036692, MT036693, MT036694, MT036695, MT036696, MT036697, MT036698, MT036699, MT036701, MT036702, MT036703, MT036704, MT036705, MT036706, MT036707, MT036708, MT036709, MT036710, MT036711, MT036712, MT036713, MT036714, MT036715, MT036716, MT036717, MT036718, MT036719, MT036720, MT036721, MT036722, MT036723, MT036724, MT036725, MT036726, MT036727, MT036728, MT036729, MT036730, MT036731, MT036732, MT036733, MT036734, MT036735, MT036736, MT036737, MT036738, MT036739, MT036740, MT036741, MT036742, MT036743, MT036744, MT036745, MT036746, MT036747, MT036748, MT036749, MT036750, MT036751, MT036752, MT036753, MT036754, MT036755, MT036756, and MT036757.

## Author Contributions

TK was responsible for the study design and wrote the manuscript. TK, AD, KB, MŻ, AS, SS, MB, MP, and AB-C contributed the isolation and strain growth. KB and MŻ extracted fungal DNA and prepared a library for whole-genome sequencing. TK and BB assembled the mitogenomes. BB, KM, and TM performed the mitogenome annotation. JS performed the phylogenetic analysis. AD, AS, KB, MB, MP, MŻ, and JS contributed the manuscript editing of relevant sections. JW edited supplementary files.

## Conflict of Interest

The authors declare that the research was conducted in the absence of any commercial or financial relationships that could be construed as a potential conflict of interest.

## Footnotes

1 https://www.ncbi.nlm.nih.gov/orffinder/

2 http://megasun.bch.umontreal.ca/cgi-bin/mfannot/mfannotInterface.pl
