# Supplementary material for: Diversity of Mobile Genetic Elements in the Mitogenomes of Closely Related Fusarium culmorum and F. graminearum sensu stricto Strains and Its Implication for Diagnostic Purposes
Source: Front Microbiol. 2020 May 25;11:1002. doi: 10.3389/fmicb.2020.01002 (PMC7263005; doi:10.3389/fmicb.2020.01002)
Supplement: Supplementary file 2 [file Table_2.DOCX]

**Supplementary file 2a. Characteristics of introns and associated HEGs found in the *cob* gene**

|  |  | *cob* | | | | | | | | | | | | | | | | | |  |  | | | | | | | | | | | | | | | |
| --- | --- | --- | --- | --- | --- | --- | --- | --- | --- | --- | --- | --- | --- | --- | --- | --- | --- | --- | --- | --- | --- | --- | --- | --- | --- | --- | --- | --- | --- | --- | --- | --- | --- | --- | --- | --- |
|  |  |  | *i1a* | | |  | | *i2* |  | *i3* |  | *i4a* |  | | | *i5* |  | *i6* |  |  |  | | | | *F. cerealis* | | | | | | | | | | | |
|  |  |  | ● | | ○^3^ |  | | ● |  | ○ |  | ● |  |  |  | ● |  | ◊ |  |  |  | | | | | | | | | | | | | | | |
|  |  |  |  | |  |  | |  |  |  |  |  |  |  |  |  |  |  |  |  |  | |  |  | |  |  | | |  | | |  | |  | |
|  |  |  |  | |  |  | |  |  |  |  | *i4a* |  |  |  |  |  |  |  |  |  | |  |  | |  |  | | |  | | |  | |  | |
|  |  |  |  | |  |  | |  |  |  |  | *i4b* | |  |  |  |  |  |  |  |  | |  |  | |  |  | | |  | | |  | |  | |
|  |  |  | *i1b* | |  |  | | *i2* |  | *i3* |  | *i4c* | | |  | *i5* |  | *i6* |  |  |  | | | | *F. culmorum* | | | | | | | | | | | |
|  |  |  | ● | |  |  | | ● |  | ○ |  | ● | ●^4^ | ◊^5^ |  | ● |  | ◊ |  |  |  | | | | | | | | | | | | | | |  |
|  |  |  |  | |  |  |  | |  |  |  |  |  |  |  |  |  |  |  |  |  |  | |  | |  | | |  | | |  | |  | |  |
|  |  |  |  | |  |  |  | |  |  |  | *i4a* |  |  |  |  |  |  |  |  |  |  | |  | |  | | |  | | |  | |  | |  |
|  |  |  |  | |  |  |  | |  |  |  | *i4b* | |  |  |  |  |  |  |  |  |  | |  | |  | | |  | | |  | |  | |  |
|  | *iII^1^* |  | *i1b*^2^ | |  |  | *i2* | |  | *i3* |  | *i4c* | | |  | *i5* |  | *i6* |  |  |  | | | | *F. graminearum s.s.* | | | | | | | | | | | |
|  |  |  | ●^2^ | |  |  | ● | |  | ○ |  | ● | ●^4^ | ◊^5^ |  | ● |  | ◊ |  |  |  | | | | | | | | | | | | | | |  |
|  |  |  |  | |  |  | |  |  |  |  |  |  |  |  |  |  |  |  |  |  |  | |  | |  | |  | | |  | | |  | |  |
|  |  |  |  | |  |  | |  |  |  |  |  |  |  |  |  |  |  |  |  |  |  | |  | |  | |  | | |  | | |  | |  |
|  |  |  |  | |  |  | |  |  |  |  |  |  |  |  |  |  |  |  |  |  |  | |  | |  | |  | | |  | | |  | |  |
|  |  |  | | *i1a* | |  | | *i2* |  | *i3* |  | *i4c* | | |  | *i5* |  | *i6* |  | *i7* | | |  |  | | *F. pseudograminearum* | | | | | | | | | | |
|  |  |  | | ● | ○ |  | | ● |  | ○ |  | ● | ● | ◊ |  | ● |  | ◊ |  | ○ | ● | |  |  | |  | | | | | | | | | | |

| Intron names: *iII - i7* | |  |  |  |  |
| --- | --- | --- | --- | --- | --- |
| Intron type: | IA | IB | IC1 | ID | HEG type: ● - LAGLIDADG, ○ - GIY-YIG, ◊ – unknown type |
| ^1^ – present in two strains of *F. graminearum* s.s., ^2^ – absent in 76 strains of *F. graminearum* s.s., ^3^ – absent in 5 strains of *F. cerealis,* ^4^ – absent in 26 strains of *F. culmorum* and 75 strains of *F. graminearum* s.s., ^5^ – absent in 27 strains of *F. culmorum* and 93 strains of *F. graminearum* s.s. | | | | | |

**Supplementary file 2b. Distribution of HEG homologs in the GenBank protein collection**

| Host | Intron and HEG | | | | | | | | | | |
| --- | --- | --- | --- | --- | --- | --- | --- | --- | --- | --- | --- |
|  | *i1a/b* | | *i2* | *i3* | *i4a/b/c* | | | *i5* | *i6* | *i7* | |
|  | ● | ○ | ● | ○ | ● | ● | ◊ | ● | ◊ | ○ | ● |
| *Fusarium cerealis* |  |  |  |  |  |  |  |  |  |  |  |
| *Fusarium culmorum* |  |  |  |  |  |  |  |  |  |  |  |
| *Fusarium graminearum s.s.* |  |  |  |  |  |  |  |  |  |  |  |
| *Fusarium pseudograminearum* |  |  |  |  |  |  |  |  |  |  |  |
| *Fusarium acaciae-mearnsii* |  |  |  |  |  |  |  |  |  |  |  |
| *Fusarium acuminatum* |  |  |  |  |  |  |  |  |  |  |  |
| *Fusarium circinatum* |  |  |  |  |  |  |  |  |  |  |  |
| *Fusarium fujikuroi* |  |  |  |  |  |  |  |  |  |  |  |
| *Fusarium gerlachii* |  |  |  |  |  |  |  |  |  |  |  |
| *Fusarium oxysporum* |  |  |  |  |  |  |  |  |  |  |  |
| *Agrocybe chaxingu* |  |  |  |  |  |  |  |  |  |  |  |
| *Amanita thiersii* |  |  |  |  |  |  |  |  |  |  |  |
| *Annulohypoxylon stygium* |  |  |  |  |  |  |  |  |  |  |  |
| *Arthrobotrys musiformis* |  |  |  |  |  |  |  |  |  |  |  |
| *Aspergillus brasiliensis* |  |  |  |  |  |  |  |  |  |  |  |
| *Aspergillus fisheri* |  |  |  |  |  |  |  |  |  |  |  |
| *Aspergillus nidulans* |  |  |  |  |  |  |  |  |  |  |  |
| *Aspergillus pseudoglaucus* |  |  |  |  |  |  |  |  |  |  |  |
| *Aspergillus ruber* |  |  |  |  |  |  |  |  |  |  |  |
| *Beauveria bassiana* |  |  |  |  |  |  |  |  |  |  |  |
| *Beauveria caledonica* |  |  |  |  |  |  |  |  |  |  |  |
| *Beauveria malawiensis* |  |  |  |  |  |  |  |  |  |  |  |
| *Bipolaris cookei* |  |  |  |  |  |  |  |  |  |  |  |
| *Bipolaris maydis* |  |  |  |  |  |  |  |  |  |  |  |
| *Bipolaris oryzae* |  |  |  |  |  |  |  |  |  |  |  |
| *Botrytis cinerea* |  |  |  |  |  |  |  |  |  |  |  |
| *Cantharellus appalachiensis* |  |  |  |  |  |  |  |  |  |  |  |
| *Cantharellus cibarius* |  |  |  |  |  |  |  |  |  |  |  |
| *Cenococcum geophilum* |  |  |  |  |  |  |  |  |  |  |  |
| *Ceratocystis cacaofunesta* |  |  |  |  |  |  |  |  |  |  |  |
| *Cercospora zeae-maydis* |  |  |  |  |  |  |  |  |  |  |  |
| *Chrysoporthe austroafricana* |  |  |  |  |  |  |  |  |  |  |  |
| *Chrysoporthe deuterocubensis* |  |  |  |  |  |  |  |  |  |  |  |
| *Colletotrichum lindemuthianum* |  |  |  |  |  |  |  |  |  |  |  |
| *Conidiobolus heterosporus* |  |  |  |  |  |  |  |  |  |  |  |
| *Cordyceps cicadae* |  |  |  |  |  |  |  |  |  |  |  |
| *Cordyceps militaris* |  |  |  |  |  |  |  |  |  |  |  |
| *Cryphonectria parasitica* |  |  |  |  |  |  |  |  |  |  |  |
| *Curvularia trifolii* |  |  |  |  |  |  |  |  |  |  |  |
| *Dactylella sp.* |  |  |  |  |  |  |  |  |  |  |  |
| *Dactylella tenuis* |  |  |  |  |  |  |  |  |  |  |  |
| *Didymella exigua* |  |  |  |  |  |  |  |  |  |  |  |
| *Drechslerella brochopaga* |  |  |  |  |  |  |  |  |  |  |  |
| *Epichloe festucae* |  |  |  |  |  |  |  |  |  |  |  |
| *Epichloe hybrida* |  |  |  |  |  |  |  |  |  |  |  |
| *Epichloe typhina* |  |  |  |  |  |  |  |  |  |  |  |
| *Fomitiporia mediterranea* |  |  |  |  |  |  |  |  |  |  |  |
| *Golovinomyces cichoracearum* |  |  |  |  |  |  |  |  |  |  |  |
| *Hirsutella rhossiliensis* |  |  |  |  |  |  |  |  |  |  |  |
| *Histoplasma capsulatum* |  |  |  |  |  |  |  |  |  |  |  |
| *Hypomyces aurantius* |  |  |  |  |  |  |  |  |  |  |  |
| *Hypoxylon sp.* |  |  |  |  |  |  |  |  |  |  |  |
| *Juglanconis juglandina* |  |  |  |  |  |  |  |  |  |  |  |
| *Juglanconis oblonga* |  |  |  |  |  |  |  |  |  |  |  |
| *Juglanconis sp.* |  |  |  |  |  |  |  |  |  |  |  |
| *Laccaria amethystina* |  |  |  |  |  |  |  |  |  |  |  |
| *Lepidopterella palustris* |  |  |  |  |  |  |  |  |  |  |  |
| *Leptographium lundbergii* |  |  |  |  |  |  |  |  |  |  |  |
| *Leptographium truncatum* |  |  |  |  |  |  |  |  |  |  |  |
| *Leptosphaeria maculans* |  |  |  |  |  |  |  |  |  |  |  |
| *Moniliophthora roreri* |  |  |  |  |  |  |  |  |  |  |  |
| *Morchella importuna* |  |  |  |  |  |  |  |  |  |  |  |
| *Myochromella boudieri* |  |  |  |  |  |  |  |  |  |  |  |
| *Neurospora crassa* |  |  |  |  |  |  |  |  |  |  |  |
| *Oidium neolycopersici* |  |  |  |  |  |  |  |  |  |  |  |
| *Ophiocordyceps sinensis* |  |  |  |  |  |  |  |  |  |  |  |
| *Ophiognomonia clavigignenti-juglandacearum* |  |  |  |  |  |  |  |  |  |  |  |
| *Ophiostoma ulmi* |  |  |  |  |  |  |  |  |  |  |  |
| *Parastagonospora nodorum* |  |  |  |  |  |  |  |  |  |  |  |
| *Pestalotiopsis fici* |  |  |  |  |  |  |  |  |  |  |  |
| *Pithomyces chartarum* |  |  |  |  |  |  |  |  |  |  |  |
| *Podospora anserina* |  |  |  |  |  |  |  |  |  |  |  |
| *Podospora comata* |  |  |  |  |  |  |  |  |  |  |  |
| *Podospora curvicolla* |  |  |  |  |  |  |  |  |  |  |  |
| *Porodaedalea pini* |  |  |  |  |  |  |  |  |  |  |  |
| *Postia placenta* |  |  |  |  |  |  |  |  |  |  |  |
| *Pseudocercospora fijiensis* |  |  |  |  |  |  |  |  |  |  |  |
| *Rickettsiales bacterium* |  |  |  |  |  |  |  |  |  |  |  |
| *Rutstroemia sp.* |  |  |  |  |  |  |  |  |  |  |  |
| *Scatalidium sp.* |  |  |  |  |  |  |  |  |  |  |  |
| *Sclerotinia borealis* |  |  |  |  |  |  |  |  |  |  |  |
| *Sordaria macrospora* |  |  |  |  |  |  |  |  |  |  |  |
| *Staphylococcus aureus* |  |  |  |  |  |  |  |  |  |  |  |
| *Stemphylium lycopersici* |  |  |  |  |  |  |  |  |  |  |  |
| *Talaromyces marneffei* |  |  |  |  |  |  |  |  |  |  |  |
| *Talaromyces stipitatus* |  |  |  |  |  |  |  |  |  |  |  |
| *Tephrocybe rancida* |  |  |  |  |  |  |  |  |  |  |  |
| *Tolypocladium ophioglossoides* |  |  |  |  |  |  |  |  |  |  |  |
| *Trichoderma reesei* |  |  |  |  |  |  |  |  |  |  |  |
| *Tricholoma bakamatsutake* |  |  |  |  |  |  |  |  |  |  |  |

| Identity | | |  |  |  |
| --- | --- | --- | --- | --- | --- |
| 90-100% | 80-90% | 70-80% | 60-70% |  |  |
|  |  |  |  |  |  |
| Hits were retained only if they had an e-value cut off lower than 0.001 and which covered at least 70% of the query sequence with >60% identity. | | | | |  |
| HEG type: ● - LAGLIDADG, ○ - GIY-YIG | | | | |  |
