# Supplementary material for: Diversity of Mobile Genetic Elements in the Mitogenomes of Closely Related Fusarium culmorum and F. graminearum sensu stricto Strains and Its Implication for Diagnostic Purposes
Source: Front Microbiol. 2020 May 25;11:1002. doi: 10.3389/fmicb.2020.01002 (PMC7263005; doi:10.3389/fmicb.2020.01002)
Supplement: Supplementary file 3 [file Table_3.DOCX]

**Supplementary file 3a. Characteristics of introns and associated HEGs found in the *cox2 and cox3* genes**

| *cox2* | | | | | | | | | | | | |  |  | *cox3* | | | | | | |  |  |  |  | |  | | |  |  |  |  |
| --- | --- | --- | --- | --- | --- | --- | --- | --- | --- | --- | --- | --- | --- | --- | --- | --- | --- | --- | --- | --- | --- | --- | --- | --- | --- | --- | --- | --- | --- | --- | --- | --- | --- |
|  |  |  |  |  |  |  |  |  | *i4* |  | *i5* |  |  |  |  | *i1* |  | *i2* |  | *i3* |  |  |  | *i5* |  |  | *F. cerealis* | | | | | | |
|  |  |  |  |  |  |  |  |  | ○ |  | ○ |  |  |  |  | ● |  | ● |  | ● |  |  |  |  |  | |  |  |  |  |  |  |  |
|  |  |  |  |  |  |  |  |  |  |  |  |  |  |  |  |  |  |  |  |  |  |  |  |  |  | |  |  |  |  |  |  |  |
|  |  |  |  |  |  |  |  |  |  |  |  |  |  |  |  |  |  |  |  |  |  |  |  |  |  | |  |  |  |  |  |  |  |
|  |  |  |  |  |  |  |  |  |  |  |  |  |  |  |  |  |  |  |  |  |  |  |  |  |  | |  |  |  |  |  |  |  |
|  | *i1* |  | *i2* |  | *i3a* | | |  | *i4^3^* |  | *i5* |  |  |  |  | *i1* |  | *i2* |  |  |  | *i4* |  |  |  | | *F. culmorum* | | | | | | |
|  | ● |  | ○ |  | ● | ● | ● |  | ○^3^ |  | ○ |  |  |  |  | ● |  | ● |  |  |  | ● |  |  |  | |  |  |  |  |  |  |  |
|  |  |  |  |  |  |  |  |  |  |  |  |  |  |  |  |  |  |  |  |  |  |  |  |  |  | |  |  |  |  |  |  |  |
|  |  |  |  |  |  |  |  |  |  |  |  |  |  |  |  |  |  |  |  |  |  |  |  |  |  | |  |  |  |  |  |  |  |
|  |  |  |  |  |  |  |  |  |  |  |  |  |  |  |  |  |  |  |  |  |  |  |  |  |  | |  |  |  |  |  |  |  |
|  | *i1* |  | *i2^1^* |  | *i3b*^2^ | | |  | *i4^3^* |  | *i5* |  |  |  |  | *i1* |  | *i2* |  |  |  |  |  |  |  | | *F. graminearum s.s.* | | | | | | |
|  | ● |  | ○^1^ |  | ●^2^ |  | ●^2^ |  | ○^3^ |  | ○ |  |  |  |  | ● |  | ● |  |  |  |  |  |  |  | |  |  |  |  |  |  |  |
|  |  |  |  |  |  |  |  |  |  |  |  |  |  |  |  |  |  |  |  |  |  |  |  |  |  | |  |  |  |  |  |  |  |
|  |  |  |  |  |  |  |  |  |  |  |  |  |  |  |  |  |  |  |  |  |  |  |  |  |  | |  |  |  |  |  |  |  |
|  |  |  |  |  |  |  |  |  |  |  |  |  |  |  |  |  |  |  |  |  |  |  |  |  |  | |  |  |  |  |  |  |  |
|  |  |  |  |  |  |  |  |  | *i4* |  | *i5* |  |  |  |  | *i1* |  | *i2* |  | *i3* |  |  |  | *i5* |  | | *F. pseudograminearum* | | | | | | |
|  |  |  |  |  |  |  |  |  | ○ |  | ○ |  |  |  |  | ● |  | ● |  | ● |  |  |  |  |  | |  |  |  |  |  |  |  |

| Intron names: *i1 – i5* | | |  |  |  |  | |
| --- | --- | --- | --- | --- | --- | --- | --- |
| Intron type: | I | IA | IB | IC1 | IC2 | ID | HEG type: ● - LAGLIDADG, ○ - GIY-YIG |
| ^1^ – absent in 37 strains of *F. graminearum* s.s., ^2^ – present in single strain of *F. graminearum* s.s., ^3^ – absent in 27 strains of *F. culmorum* and 90 strains of *F. graminearum* s.s. | | | | | | | |

**Supplementary file 3b. Distribution of HEG homologs in the GenBank protein collection**

| Host |  | Intron and HEG | | | | | | | | | | | |
| --- | --- | --- | --- | --- | --- | --- | --- | --- | --- | --- | --- | --- | --- |
|  |  | *cox2* | | | | | | |  | *cox3* | | | |
|  | *i1a* | | *i2* | *i3ab* | | | *i4* | *i5* |  | *i1* | *i2* | *i3* | *i4* |
|  | ● | | ○ | ● | ● | ● | ○ | ○ |  | ● | ● | ● | ● |
| *Fusarium cerealis* |  | |  |  |  |  |  |  |  |  |  |  |  |
| *Fusarium culmorum* |  | |  |  |  |  |  |  |  |  |  |  |  |
| *Fusarium graminearum s.s.* |  | |  |  |  |  |  |  |  |  |  |  |  |
| *Fusarium pseudograminearum* |  | |  |  |  |  |  |  |  |  |  |  |  |
| *Fusarium bambusae* |  | |  |  |  |  |  |  |  |  |  |  |  |
| *Fusarium circinatum* |  | |  |  |  |  |  |  |  |  |  |  |  |
| *Fusarium gerlachii* |  | |  |  |  |  |  |  |  |  |  |  |  |
| *Fusarium solani* |  | |  |  |  |  |  |  |  |  |  |  |  |
| *Fusarium temperatum* |  | |  |  |  |  |  |  |  |  |  |  |  |
| *Agaricus bisporus* |  | |  |  |  |  |  |  |  |  |  |  |  |
| *Annulohypoxylon stygium* |  | |  |  |  |  |  |  |  |  |  |  |  |
| *Arthrobotrys oligospora* |  | |  |  |  |  |  |  |  |  |  |  |  |
| *Aspergillus pseudoglaucus* |  | |  |  |  |  |  |  |  |  |  |  |  |
| *Beauveria bassiana* |  | |  |  |  |  |  |  |  |  |  |  |  |
| *Beauveria caledonica* |  | |  |  |  |  |  |  |  |  |  |  |  |
| *Beauveria malawiensis* |  | |  |  |  |  |  |  |  |  |  |  |  |
| *Bipolaris cookei* |  | |  |  |  |  |  |  |  |  |  |  |  |
| *Bipolaris maydis* |  | |  |  |  |  |  |  |  |  |  |  |  |
| *Bipolaris oryzae* |  | |  |  |  |  |  |  |  |  |  |  |  |
| *Botrytis cinerea* |  | |  |  |  |  |  |  |  |  |  |  |  |
| *Ceratocystis albifundus* |  | |  |  |  |  |  |  |  |  |  |  |  |
| *Ceratocystis cacaofunesta* |  | |  |  |  |  |  |  |  |  |  |  |  |
| *Ceratocystis fimbriata* |  | |  |  |  |  |  |  |  |  |  |  |  |
| *Chrysoporthe austroafricana* |  | |  |  |  |  |  |  |  |  |  |  |  |
| *Chrysoporthe cubensis* |  | |  |  |  |  |  |  |  |  |  |  |  |
| *Chrysoporthe deuterocubensis* |  | |  |  |  |  |  |  |  |  |  |  |  |
| *Coniothyrium glycines* |  | |  |  |  |  |  |  |  |  |  |  |  |
| *Cordyceps cicadae* |  | |  |  |  |  |  |  |  |  |  |  |  |
| *Cordyceps militaris* |  | |  |  |  |  |  |  |  |  |  |  |  |
| *Cryphonectria parasitica* |  | |  |  |  |  |  |  |  |  |  |  |  |
| *Curvularia trifolii* |  | |  |  |  |  |  |  |  |  |  |  |  |
| *Dactylella sp.* |  | |  |  |  |  |  |  |  |  |  |  |  |
| *Epichloe festucae* |  | |  |  |  |  |  |  |  |  |  |  |  |
| *Epichloe typhina* |  | |  |  |  |  |  |  |  |  |  |  |  |
| *Ganoderma meredithae* |  | |  |  |  |  |  |  |  |  |  |  |  |
| *Ganoderma sinense* |  | |  |  |  |  |  |  |  |  |  |  |  |
| *Ganoderma sp.* |  | |  |  |  |  |  |  |  |  |  |  |  |
| *Golovinomyces cichoracearum* |  | |  |  |  |  |  |  |  |  |  |  |  |
| *Hypomyces aurantius* |  | |  |  |  |  |  |  |  |  |  |  |  |
| *Inonotus obliquus* |  | |  |  |  |  |  |  |  |  |  |  |  |
| *Lactifluus hygrophoroides* |  | |  |  |  |  |  |  |  |  |  |  |  |
| *Magnusiomyces ingens* |  | |  |  |  |  |  |  |  |  |  |  |  |
| *Monilinia fructicola* |  | |  |  |  |  |  |  |  |  |  |  |  |
| *Morchella importuna* |  | |  |  |  |  |  |  |  |  |  |  |  |
| *Ophiocordyceps sinensis* |  | |  |  |  |  |  |  |  |  |  |  |  |
| *Ophiostoma ulmi* |  | |  |  |  |  |  |  |  |  |  |  |  |
| *Pestalotiopsis fici* |  | |  |  |  |  |  |  |  |  |  |  |  |
| *Podospora anserina* |  | |  |  |  |  |  |  |  |  |  |  |  |
| *Podospora comata* |  | |  |  |  |  |  |  |  |  |  |  |  |
| *Postia placenta* |  | |  |  |  |  |  |  |  |  |  |  |  |
| *Rhynchosporium agropyri* |  | |  |  |  |  |  |  |  |  |  |  |  |
| *Rhynchosporium commune* |  | |  |  |  |  |  |  |  |  |  |  |  |
| *Rhynchosporium secalis* |  | |  |  |  |  |  |  |  |  |  |  |  |
| *Rickettsiales bacterium* |  | |  |  |  |  |  |  |  |  |  |  |  |
| *Scatalidium sp.* |  | |  |  |  |  |  |  |  |  |  |  |  |
| *Sclerotninia sclerotiorum* |  | |  |  |  |  |  |  |  |  |  |  |  |
| *Stemphylium lycopersici* |  | |  |  |  |  |  |  |  |  |  |  |  |
| *Trichoderma asperellum* |  | |  |  |  |  |  |  |  |  |  |  |  |
| *Trichoderma hamatum* |  | |  |  |  |  |  |  |  |  |  |  |  |

| Identity | | |  |  |  |
| --- | --- | --- | --- | --- | --- |
| 90-100% | 80-90% | 70-80% | 60-70% |  |  |
|  |  |  |  |  |  |
| Hits were retained only if they had an e-value cut off lower than 0.001 and which covered at least 70% of the query sequence with >60% identity. | | | | |  |
| HEG type: ● - LAGLIDADG, ○ - GIY-YIG | | | | |  |
