# Supplementary material for: Diversity of Mobile Genetic Elements in the Mitogenomes of Closely Related Fusarium culmorum and F. graminearum sensu stricto Strains and Its Implication for Diagnostic Purposes
Source: Front Microbiol. 2020 May 25;11:1002. doi: 10.3389/fmicb.2020.01002 (PMC7263005; doi:10.3389/fmicb.2020.01002)
Supplement: Supplementary file 4 [file Table_4.DOCX]

**Supplementary file 4a. Characteristics of introns and associated HEGs found in the *cox1* gene**

| *cox1* | | | | | | | | | | | | | | | | | | | | | | | | | | | | | | | |  |  |  |  |  |  |  |
| --- | --- | --- | --- | --- | --- | --- | --- | --- | --- | --- | --- | --- | --- | --- | --- | --- | --- | --- | --- | --- | --- | --- | --- | --- | --- | --- | --- | --- | --- | --- | --- | --- | --- | --- | --- | --- | --- | --- |
|  | *i1a* |  |  |  |  |  |  |  | *i5* |  | *i6* |  | *i7* |  | *i8* |  | *i9* |  | *i10* |  |  |  | *i11b* |  | *i12* | |  | *i13* |  | *i14* |  |  |  |  |  |  |  | *F. cerealis* |
|  | ● |  |  |  |  |  |  |  | ● |  | ● |  | ● |  | ● |  | ● |  | ● |  |  |  | ● |  | ○ | ○ |  | ● |  | ○ |  |  |  |  |  |  |  |  |
|  |  |  |  |  |  |  |  |  |  |  |  |  |  |  |  |  |  |  |  |  |  |  |  |  |  |  |  |  |  |  |  |  |  |  |  |  |  |  |
|  |  |  |  |  |  |  |  |  |  |  |  |  |  |  |  |  |  |  |  |  |  |  |  |  |  |  |  |  |  |  |  |  |  |  |  |  |  |  |
|  |  |  |  |  |  |  |  |  |  |  |  |  |  |  |  |  |  |  |  |  |  |  |  |  |  |  |  |  |  |  |  |  |  |  |  |  |  |  |
|  | *i1b* |  |  |  |  |  | *i4* |  | *i5* |  | *i6* |  | *i7* |  | *i8* |  | *i9* |  | *i10* |  |  |  | *i11b* |  | *i12* | |  | *i13* |  | *i14* |  | *i15^2^* |  |  |  |  |  | *F. culmorum* |
|  | ○ |  |  |  |  |  | ● |  | ● |  | ● |  | ● |  | ● |  | ● |  | ● |  |  |  | ● |  | ○ | ○ |  | ● |  | ○ |  | ○^2^ |  |  |  |  |  |  |
|  |  |  |  |  |  |  |  |  |  |  |  |  |  |  |  |  |  |  |  |  |  |  |  |  |  |  |  |  |  |  |  |  |  |  |  |  |  |  |
|  |  |  |  |  |  |  |  |  |  |  |  |  |  |  |  |  |  |  |  |  |  |  |  |  |  |  |  |  |  |  |  |  |  |  |  |  |  |  |
|  |  |  |  |  |  |  |  |  |  |  |  |  |  |  |  |  |  |  |  |  |  |  |  |  |  |  |  |  |  |  |  |  |  |  |  |  |  |  |
|  | *i1b* |  |  |  |  |  | *i4* |  | *i5* |  | *i6* |  | *i7* |  | *i8* |  | *i9* |  | *i10* |  | *i11a* | | |  | *i12* | |  | *i13* |  | *i14* |  | *i15^2^* |  |  |  |  |  | *F. gramine- -arum s.s.* |
|  | ○ |  |  |  |  |  | ● |  | ● |  | ● |  | ● |  | ● |  | ● |  | ● |  | ●^1^ | | ● |  | ○ | ○ |  | ● |  | ○ |  | ○^2^ |  |  |  |  |  |  |
|  |  |  |  |  |  |  |  |  |  |  |  |  |  |  |  |  |  |  |  |  |  |  |  |  |  |  |  |  |  |  |  |  |  |  |  |  |  |  |
|  |  |  |  |  |  |  |  |  |  |  |  |  |  |  |  |  |  |  |  |  |  |  |  |  |  |  |  |  |  |  |  |  |  |  |  |  |  |  |
|  |  |  |  |  |  |  |  |  |  |  |  |  |  |  |  |  |  |  |  |  |  |  |  |  |  |  |  |  |  |  |  |  |  |  |  |  |  |  |
|  | *i1b* |  | *i2* |  | *i3* |  |  |  | *i5* |  | *i6* |  | *i7* |  | *i8* |  | *i9* |  | *i10* |  |  |  | *i11b* |  | *i12* | |  | *i13* |  | *i14* |  | *i15* |  | *i16* |  | *i17* |  | *F. pseudo-gramine-arum* |
|  | ○ |  | ● |  | ● |  |  |  | ● |  | ● |  | ● |  | ● |  | ● |  | ● |  |  |  | ● |  | ○ | ○ |  | ● |  | ○ |  | ○ |  | ○ |  | - |  |  |

| Intron names: *i1 – i17* | |  |  |  |  | |
| --- | --- | --- | --- | --- | --- | --- |
| Intron type: | I | IA | IB | D |  | HEG type: ● - LAGLIDADG, ○ - GIY-YIG |
| ^1^ – present in single strain of *F. graminearum* s.s. , ^2^ – absent in all but one strain of *F. culmorum* and in 12 strains of *F. graminearum* s.s., | | | | | | |

| **Supplementary file 4b. Distribution of HEG homologs in the GenBank protein collection** | | | | | | | | | | | | | | | | | | | |
| --- | --- | --- | --- | --- | --- | --- | --- | --- | --- | --- | --- | --- | --- | --- | --- | --- | --- | --- | --- |
| Host | Intron and HEG | | | | | | | | | | | | | | | | | | |
|  | *i1a* | *i1b* | *i2* | *i3* | *i4* | *i5* | *i6* | *i7* | *i8* | *i9* | *i10* | *i11a* | *i11b* | *i12* | | *i13* | *i14* | *i15* | *i16* |
|  | ● | ○ | ● | ● | ● | ● | ● | ● | ● | ● | ● | ● | ● | ○ | ○ | ● | ○ | ○ | ○ |
| *Fusarium cerealis* |  |  |  |  |  |  |  |  |  |  |  |  |  |  |  |  |  |  |  |
| *Fusarium culmorum* |  |  |  |  |  |  |  |  |  |  |  |  |  |  |  |  |  |  |  |
| *Fusarium graminearum s.s.* |  |  |  |  |  |  |  |  |  |  |  |  |  |  |  |  |  |  |  |
| *Fusarium pseudograminearum* |  |  |  |  |  |  |  |  |  |  |  |  |  |  |  |  |  |  |  |
| *Fusarium acuminatum* |  |  |  |  |  |  |  |  |  |  |  |  |  |  |  |  |  |  |  |
| *Fusarium bambusae* |  |  |  |  |  |  |  |  |  |  |  |  |  |  |  |  |  |  |  |
| *Fusarium circinatum* |  |  |  |  |  |  |  |  |  |  |  |  |  |  |  |  |  |  |  |
| *Fusarium gerlachii* |  |  |  |  |  |  |  |  |  |  |  |  |  |  |  |  |  |  |  |
| *Fusarium mangiferae* |  |  |  |  |  |  |  |  |  |  |  |  |  |  |  |  |  |  |  |
| *Fusarium solani* |  |  |  |  |  |  |  |  |  |  |  |  |  |  |  |  |  |  |  |
| *Fusarium temperatum* |  |  |  |  |  |  |  |  |  |  |  |  |  |  |  |  |  |  |  |
| *Fusarium venenatum* |  |  |  |  |  |  |  |  |  |  |  |  |  |  |  |  |  |  |  |
| *Fusarium verticillioides* |  |  |  |  |  |  |  |  |  |  |  |  |  |  |  |  |  |  |  |
| *Agaricus bisporus* |  |  |  |  |  |  |  |  |  |  |  |  |  |  |  |  |  |  |  |
| *Agaricus devoniensis* |  |  |  |  |  |  |  |  |  |  |  |  |  |  |  |  |  |  |  |
| *Agrocybe aegerita* |  |  |  |  |  |  |  |  |  |  |  |  |  |  |  |  |  |  |  |
| *Annulohypoxylon stygium* |  |  |  |  |  |  |  |  |  |  |  |  |  |  |  |  |  |  |  |
| *Aplosporella prunicola* |  |  |  |  |  |  |  |  |  |  |  |  |  |  |  |  |  |  |  |
| *Armanita muscaria* |  |  |  |  |  |  |  |  |  |  |  |  |  |  |  |  |  |  |  |
| *Armanita phalloides* |  |  |  |  |  |  |  |  |  |  |  |  |  |  |  |  |  |  |  |
| *Armillaria solidipes* |  |  |  |  |  |  |  |  |  |  |  |  |  |  |  |  |  |  |  |
| *Arthrobotrys musiformis* |  |  |  |  |  |  |  |  |  |  |  |  |  |  |  |  |  |  |  |
| *Arthrobotrys oligospora* |  |  |  |  |  |  |  |  |  |  |  |  |  |  |  |  |  |  |  |
| *Aspergillus fischeri* |  |  |  |  |  |  |  |  |  |  |  |  |  |  |  |  |  |  |  |
| *Aspergillus fumigatus* |  |  |  |  |  |  |  |  |  |  |  |  |  |  |  |  |  |  |  |
| *Aspergillus nidulans* |  |  |  |  |  |  |  |  |  |  |  |  |  |  |  |  |  |  |  |
| *Aspergillus pseudoglaucus* |  |  |  |  |  |  |  |  |  |  |  |  |  |  |  |  |  |  |  |
| *Aspergillus ruber* |  |  |  |  |  |  |  |  |  |  |  |  |  |  |  |  |  |  |  |
| *Atta colombica* |  |  |  |  |  |  |  |  |  |  |  |  |  |  |  |  |  |  |  |
| *Beauveria bassiana* |  |  |  |  |  |  |  |  |  |  |  |  |  |  |  |  |  |  |  |
| *Beauveria brongniartii* |  |  |  |  |  |  |  |  |  |  |  |  |  |  |  |  |  |  |  |
| *Beauveria caledonica* |  |  |  |  |  |  |  |  |  |  |  |  |  |  |  |  |  |  |  |
| *Beauveria malawiensis* |  |  |  |  |  |  |  |  |  |  |  |  |  |  |  |  |  |  |  |
| *Bipolaris cookei* |  |  |  |  |  |  |  |  |  |  |  |  |  |  |  |  |  |  |  |
| *Bipolaris oryzae* |  |  |  |  |  |  |  |  |  |  |  |  |  |  |  |  |  |  |  |
| *Botrytis cinerea* |  |  |  |  |  |  |  |  |  |  |  |  |  |  |  |  |  |  |  |
| *Candida oxycetoniae* |  |  |  |  |  |  |  |  |  |  |  |  |  |  |  |  |  |  |  |
| *Cantharellus appalachiensis* |  |  |  |  |  |  |  |  |  |  |  |  |  |  |  |  |  |  |  |
| *Ceratocystis cacaofunesta* |  |  |  |  |  |  |  |  |  |  |  |  |  |  |  |  |  |  |  |
| *Ceratocystis fimbriata* |  |  |  |  |  |  |  |  |  |  |  |  |  |  |  |  |  |  |  |
| *Chrysoporthe austroafricana* |  |  |  |  |  |  |  |  |  |  |  |  |  |  |  |  |  |  |  |
| *Chrysoporthe deuterocubensis* |  |  |  |  |  |  |  |  |  |  |  |  |  |  |  |  |  |  |  |
| *Cladonia apodocarpa* |  |  |  |  |  |  |  |  |  |  |  |  |  |  |  |  |  |  |  |
| *Cladonia leporina* |  |  |  |  |  |  |  |  |  |  |  |  |  |  |  |  |  |  |  |
| *Cladonia petrophila* |  |  |  |  |  |  |  |  |  |  |  |  |  |  |  |  |  |  |  |
| *Cladonia stipitata* |  |  |  |  |  |  |  |  |  |  |  |  |  |  |  |  |  |  |  |
| *Cladonia subtenuis* |  |  |  |  |  |  |  |  |  |  |  |  |  |  |  |  |  |  |  |
| *Coccocarpia palmicola* |  |  |  |  |  |  |  |  |  |  |  |  |  |  |  |  |  |  |  |
| *Conidiobolus heterosporus* |  |  |  |  |  |  |  |  |  |  |  |  |  |  |  |  |  |  |  |
| *Coniothyrium glycines* |  |  |  |  |  |  |  |  |  |  |  |  |  |  |  |  |  |  |  |
| *Cordyceps cicadae* |  |  |  |  |  |  |  |  |  |  |  |  |  |  |  |  |  |  |  |
| *Cordyceps militaris* |  |  |  |  |  |  |  |  |  |  |  |  |  |  |  |  |  |  |  |
| *Cryphonectria parasitica* |  |  |  |  |  |  |  |  |  |  |  |  |  |  |  |  |  |  |  |
| *Curvularia trifolii* |  |  |  |  |  |  |  |  |  |  |  |  |  |  |  |  |  |  |  |
| *Dactylella sp.* |  |  |  |  |  |  |  |  |  |  |  |  |  |  |  |  |  |  |  |
| *Dactylella tenuis* |  |  |  |  |  |  |  |  |  |  |  |  |  |  |  |  |  |  |  |
| *Didymella exigua* |  |  |  |  |  |  |  |  |  |  |  |  |  |  |  |  |  |  |  |
| *Diplocarpon rosae* |  |  |  |  |  |  |  |  |  |  |  |  |  |  |  |  |  |  |  |
| *Drechslerella brochopaga* |  |  |  |  |  |  |  |  |  |  |  |  |  |  |  |  |  |  |  |
| *Epichloe festucae* |  |  |  |  |  |  |  |  |  |  |  |  |  |  |  |  |  |  |  |
| *Epichloe typhina* |  |  |  |  |  |  |  |  |  |  |  |  |  |  |  |  |  |  |  |
| *Epidermophyton floccosum* |  |  |  |  |  |  |  |  |  |  |  |  |  |  |  |  |  |  |  |
| *Flammulina velutipes* |  |  |  |  |  |  |  |  |  |  |  |  |  |  |  |  |  |  |  |
| *Fomitopsis palustris* |  |  |  |  |  |  |  |  |  |  |  |  |  |  |  |  |  |  |  |
| *Ganoderma calidophilum* |  |  |  |  |  |  |  |  |  |  |  |  |  |  |  |  |  |  |  |
| *Ganoderma leucocontextum* |  |  |  |  |  |  |  |  |  |  |  |  |  |  |  |  |  |  |  |
| *Ganoderma meredithae* |  |  |  |  |  |  |  |  |  |  |  |  |  |  |  |  |  |  |  |
| *Ganoderma tsugae* |  |  |  |  |  |  |  |  |  |  |  |  |  |  |  |  |  |  |  |
| *Glarea lozoyensis* |  |  |  |  |  |  |  |  |  |  |  |  |  |  |  |  |  |  |  |
| *Hirsutella minnesotensis* |  |  |  |  |  |  |  |  |  |  |  |  |  |  |  |  |  |  |  |
| *Hirsutella rhossiliensis* |  |  |  |  |  |  |  |  |  |  |  |  |  |  |  |  |  |  |  |
| *Hirsutella thompsonii* |  |  |  |  |  |  |  |  |  |  |  |  |  |  |  |  |  |  |  |
| *Histoplasma castulatum* |  |  |  |  |  |  |  |  |  |  |  |  |  |  |  |  |  |  |  |
| *Hypomyces aurantius* |  |  |  |  |  |  |  |  |  |  |  |  |  |  |  |  |  |  |  |
| *Juglanconis juglandina* |  |  |  |  |  |  |  |  |  |  |  |  |  |  |  |  |  |  |  |
| *Juglanconis oblonga* |  |  |  |  |  |  |  |  |  |  |  |  |  |  |  |  |  |  |  |
| *Juglanconis sp.* |  |  |  |  |  |  |  |  |  |  |  |  |  |  |  |  |  |  |  |
| *Lachnellula arida* |  |  |  |  |  |  |  |  |  |  |  |  |  |  |  |  |  |  |  |
| *Lactarius deliciosus* |  |  |  |  |  |  |  |  |  |  |  |  |  |  |  |  |  |  |  |
| *Lactarius hatsudake* |  |  |  |  |  |  |  |  |  |  |  |  |  |  |  |  |  |  |  |
| *Lactarius piperatus* |  |  |  |  |  |  |  |  |  |  |  |  |  |  |  |  |  |  |  |
| *Lactifluus hygrophoroides* |  |  |  |  |  |  |  |  |  |  |  |  |  |  |  |  |  |  |  |
| *Lentinula edodes* |  |  |  |  |  |  |  |  |  |  |  |  |  |  |  |  |  |  |  |
| *Lophiostoma macrostomum* |  |  |  |  |  |  |  |  |  |  |  |  |  |  |  |  |  |  |  |
| *Macrolepiota fuliginosa* |  |  |  |  |  |  |  |  |  |  |  |  |  |  |  |  |  |  |  |
| *Madurella mycetomatic* |  |  |  |  |  |  |  |  |  |  |  |  |  |  |  |  |  |  |  |
| *Magnusiomyces capitatus* |  |  |  |  |  |  |  |  |  |  |  |  |  |  |  |  |  |  |  |
| *Marssonina brunnea* |  |  |  |  |  |  |  |  |  |  |  |  |  |  |  |  |  |  |  |
| *Monilinia fructicola* |  |  |  |  |  |  |  |  |  |  |  |  |  |  |  |  |  |  |  |
| *Moniliophthora perniciosa* |  |  |  |  |  |  |  |  |  |  |  |  |  |  |  |  |  |  |  |
| *Moniliophthora roreri* |  |  |  |  |  |  |  |  |  |  |  |  |  |  |  |  |  |  |  |
| *Morchella importuna* |  |  |  |  |  |  |  |  |  |  |  |  |  |  |  |  |  |  |  |
| *Morterella elongata* |  |  |  |  |  |  |  |  |  |  |  |  |  |  |  |  |  |  |  |
| *Mortierella verticillata* |  |  |  |  |  |  |  |  |  |  |  |  |  |  |  |  |  |  |  |
| *Myochromella boudieri* |  |  |  |  |  |  |  |  |  |  |  |  |  |  |  |  |  |  |  |
| *Ophiocordyceps sinensis* |  |  |  |  |  |  |  |  |  |  |  |  |  |  |  |  |  |  |  |
| *Ophiognomonia clavigignenti-juglandacearum* |  |  |  |  |  |  |  |  |  |  |  |  |  |  |  |  |  |  |  |
| *Ophiostoma ulmi* |  |  |  |  |  |  |  |  |  |  |  |  |  |  |  |  |  |  |  |
| *Peltigera malacea* |  |  |  |  |  |  |  |  |  |  |  |  |  |  |  |  |  |  |  |
| *Peltigera membranacea* |  |  |  |  |  |  |  |  |  |  |  |  |  |  |  |  |  |  |  |
| *Penicillium roqueforti* |  |  |  |  |  |  |  |  |  |  |  |  |  |  |  |  |  |  |  |
| *Pertusaria propinqua* |  |  |  |  |  |  |  |  |  |  |  |  |  |  |  |  |  |  |  |
| *Pestalotiopsis fici* |  |  |  |  |  |  |  |  |  |  |  |  |  |  |  |  |  |  |  |
| *Phellinus lamaoensis* |  |  |  |  |  |  |  |  |  |  |  |  |  |  |  |  |  |  |  |
| *Phlebia radiata* |  |  |  |  |  |  |  |  |  |  |  |  |  |  |  |  |  |  |  |
| *Pithomyces chartarum* |  |  |  |  |  |  |  |  |  |  |  |  |  |  |  |  |  |  |  |
| *Pleurotus citrinopileatus* |  |  |  |  |  |  |  |  |  |  |  |  |  |  |  |  |  |  |  |
| *Pleurotus ostreatus* |  |  |  |  |  |  |  |  |  |  |  |  |  |  |  |  |  |  |  |
| *Pleurotus platypus* |  |  |  |  |  |  |  |  |  |  |  |  |  |  |  |  |  |  |  |
| *Podospora anserina* |  |  |  |  |  |  |  |  |  |  |  |  |  |  |  |  |  |  |  |
| *Podospora comata* |  |  |  |  |  |  |  |  |  |  |  |  |  |  |  |  |  |  |  |
| *Porodaedalea pini* |  |  |  |  |  |  |  |  |  |  |  |  |  |  |  |  |  |  |  |
| *Postia placenta* |  |  |  |  |  |  |  |  |  |  |  |  |  |  |  |  |  |  |  |
| *Pseudogymnoascus destructans* |  |  |  |  |  |  |  |  |  |  |  |  |  |  |  |  |  |  |  |
| *Pyronema omphalodes* |  |  |  |  |  |  |  |  |  |  |  |  |  |  |  |  |  |  |  |
| *Pyrrhoderma noxium* |  |  |  |  |  |  |  |  |  |  |  |  |  |  |  |  |  |  |  |
| *Rhizopogon salebrosus* |  |  |  |  |  |  |  |  |  |  |  |  |  |  |  |  |  |  |  |
| *Rhynchosporium agropyri* |  |  |  |  |  |  |  |  |  |  |  |  |  |  |  |  |  |  |  |
| *Rhynchosporium commune* |  |  |  |  |  |  |  |  |  |  |  |  |  |  |  |  |  |  |  |
| *Rhynchosporium secalis* |  |  |  |  |  |  |  |  |  |  |  |  |  |  |  |  |  |  |  |
| *Rickettsiales bacterium* |  |  |  |  |  |  |  |  |  |  |  |  |  |  |  |  |  |  |  |
| *Russula abietina* |  |  |  |  |  |  |  |  |  |  |  |  |  |  |  |  |  |  |  |
| *Russula compacta* |  |  |  |  |  |  |  |  |  |  |  |  |  |  |  |  |  |  |  |
| *Russula foetens* |  |  |  |  |  |  |  |  |  |  |  |  |  |  |  |  |  |  |  |
| *Russula lepida* |  |  |  |  |  |  |  |  |  |  |  |  |  |  |  |  |  |  |  |
| *Russula virescens* |  |  |  |  |  |  |  |  |  |  |  |  |  |  |  |  |  |  |  |
| *Scatalidium sp.* |  |  |  |  |  |  |  |  |  |  |  |  |  |  |  |  |  |  |  |
| *Schizopora paradoxa* |  |  |  |  |  |  |  |  |  |  |  |  |  |  |  |  |  |  |  |
| *Sclerotinia bolearis* |  |  |  |  |  |  |  |  |  |  |  |  |  |  |  |  |  |  |  |
| *Sclerotinia sclerotiorum* |  |  |  |  |  |  |  |  |  |  |  |  |  |  |  |  |  |  |  |
| *Sordaria macrospora* |  |  |  |  |  |  |  |  |  |  |  |  |  |  |  |  |  |  |  |
| *Sporothrix brasiliensis* |  |  |  |  |  |  |  |  |  |  |  |  |  |  |  |  |  |  |  |
| *Sporothrix insectorum* |  |  |  |  |  |  |  |  |  |  |  |  |  |  |  |  |  |  |  |
| *Taiwanofungus camphoratus* |  |  |  |  |  |  |  |  |  |  |  |  |  |  |  |  |  |  |  |
| *Talaromyces marneffei* |  |  |  |  |  |  |  |  |  |  |  |  |  |  |  |  |  |  |  |
| *Talaromyces stipitatus* |  |  |  |  |  |  |  |  |  |  |  |  |  |  |  |  |  |  |  |
| *Termitomyces sp.* |  |  |  |  |  |  |  |  |  |  |  |  |  |  |  |  |  |  |  |
| *Tolypocladium ophioglossoides* |  |  |  |  |  |  |  |  |  |  |  |  |  |  |  |  |  |  |  |
| *Trametes hirsuta* |  |  |  |  |  |  |  |  |  |  |  |  |  |  |  |  |  |  |  |
| *Tricholoma bakamatsutake* |  |  |  |  |  |  |  |  |  |  |  |  |  |  |  |  |  |  |  |
| *Tricholoma matsutake* |  |  |  |  |  |  |  |  |  |  |  |  |  |  |  |  |  |  |  |
| *Tricholoma saponaceum* |  |  |  |  |  |  |  |  |  |  |  |  |  |  |  |  |  |  |  |
| *Tricholoma terreum* |  |  |  |  |  |  |  |  |  |  |  |  |  |  |  |  |  |  |  |
| *Tuber melanosporum* |  |  |  |  |  |  |  |  |  |  |  |  |  |  |  |  |  |  |  |
| *Zancudomyces culisetae* |  |  |  |  |  |  |  |  |  |  |  |  |  |  |  |  |  |  |  |

| Identity | | |  |  |  |
| --- | --- | --- | --- | --- | --- |
| 90-100% | 80-90% | 70-80% | 60-70% |  |  |
|  |  |  |  |  |  |
| Hits were retained only if they had an e-value cut off lower than 0.001 and which covered at least 70% of the query sequence with >70% identity. | | | | |  |
| HEG type: ● - LAGLIDADG, ○ - GIY-YIG | | | | |  |
