# Supplementary material for: Diversity of Mobile Genetic Elements in the Mitogenomes of Closely Related Fusarium culmorum and F. graminearum sensu stricto Strains and Its Implication for Diagnostic Purposes
Source: Front Microbiol. 2020 May 25;11:1002. doi: 10.3389/fmicb.2020.01002 (PMC7263005; doi:10.3389/fmicb.2020.01002)
Supplement: Supplementary file 5 [file Table_5.DOCX]

**Supplementary file 5a. Characteristics of introns and associated HEGs found in the *atp6* and *atp9* genes**

| Intron and HEG | | | | | | | | | | | | | | | | | | | | | | | | | | | | Host |
| --- | --- | --- | --- | --- | --- | --- | --- | --- | --- | --- | --- | --- | --- | --- | --- | --- | --- | --- | --- | --- | --- | --- | --- | --- | --- | --- | --- | --- |
| *atp6* | | | | | | | | | | | |  | | | | | | *atp9* | | | | | | | | | |  |
|  |  | | *i1a* | |  | |  | |  | | |  | | | | | |  | | | *i1* | | |  | |  | | *F. cerealis* |
|  |  | | ● | |  | |  | |  | | |  | | |  | | |  | | | ○ | | |  | |  | |  |
|  |  | |  | |  | |  | |  | | |  | | |  | | |  | | |  | | |  | |  | |  |
|  |  | |  | |  | |  | |  | | |  | | |  | | |  | | |  | | |  | |  | |  |
|  |  | |  | |  | |  | |  | | |  | | |  | | |  | | |  | | | | |  | |  |
|  |  | | *i1a* | |  | |  | |  | | |  | | | | | |  | | | *i1* | | |  | |  | | *F. culmorum* |
|  |  | | ● | |  | |  | |  | | |  | | |  | | |  | | | ○ | | |  | |  | |  |
|  |  | |  | |  | |  | |  | |  | | |  | | |  | | |  | | |  | | |  | |  |
|  |  | |  | |  | |  | |  | |  | | |  | | |  | | |  | | |  | | |  | |  |
|  |  | |  | |  | |  | |  | |  | | |  | | |  | | |  | | | | | |  | |  |
|  |  | *i1a* | | |  | |  | |  | |  | | | | | |  | | | *i1* | | |  | | |  | | *F. graminearum s.s.* |
|  |  | | ● | |  | |  | |  | |  | | |  | | |  | | | ○ | | |  | | |  | |  |
|  |  |  | |  | |  | |  | |  | | |  | | |  | | |  | | |  | | |  | |  | |
|  |  |  | |  | |  | |  | |  | | |  | | |  | | |  | | |  | | |  | |  | |
|  |  |  | |  | |  | |  | |  | | |  | | |  | | |  | | |  | | |  | |  | |
|  | *i1b* | | |  | | *i2* | |  | |  | | | | | |  | | | *i1* | | |  | | |  | | *F. pseudograminearum* | |
|  | ● | ● | |  | | ○ | |  | |  | | |  | | |  | | | ○ | | |  | | |  | |  |  |

| Intron names: *i1a – i2* | |  |  |  |  |
| --- | --- | --- | --- | --- | --- |
| Intron type: | IA | IB | IC2 |  | HEG type: ● - LAGLIDADG, ○ - GIY-YIG |

**Supplementary file 5b. Distribution of HEG homologs in the GenBank protein collection**

| Host |  | Intron and HEG | | | |
| --- | --- | --- | --- | --- | --- |
|  | atp6 | | |  | atp9 |
|  | *i1a* | *i1b* | *i2* |  | *i1* |
|  | ● | ● | ○ |  | ○ |
| *F. cerealis* |  |  |  |  |  |
| *F. culmoum* |  |  |  |  |  |
| *F. graminearum s.s.* |  |  |  |  |  |
| *F. pseudograminearum* |  |  |  |  |  |
| *Fusarium gerlachii* |  |  |  |  |  |
| *Fusarium oxysporum* |  |  |  |  |  |
| *Fusarium solani* |  |  |  |  |  |
| *Annulohypoxylon stygium* |  |  |  |  |  |
| *Bipolaris cookei* |  |  |  |  |  |
| *Chrysoporthe austroafricana* |  |  |  |  |  |
| *Cordyceps cicadae* |  |  |  |  |  |
| *Cryphonectria parasitica* |  |  |  |  |  |
| *Neurospora crassa* |  |  |  |  |  |
| *Ophiocordyceps sinensis* |  |  |  |  |  |
| *Pestalotiopsis fici* |  |  |  |  |  |
| *Pyronema omphalodes* |  |  |  |  |  |
| *Scytalidium sp.* |  |  |  |  |  |
| *Sordaria macrospora* |  |  |  |  |  |
| *Trichoderma reesei* |  |  |  |  |  |

| Identity | | |  |  |  |
| --- | --- | --- | --- | --- | --- |
| 90-100% | 80-90% | 70-80% | 60-70% |  |  |
|  |  |  |  |  |  |
| Hits were retained only if they had an e-value cut off lower than 0.001 and which covered at least 70% of the query sequence with >60% identity. | | | | |  |
| HEG type: ● - LAGLIDADG, ○ - GIY-YIG | | | | |  |
