# Supplementary material for: Diversity of Mobile Genetic Elements in the Mitogenomes of Closely Related Fusarium culmorum and F. graminearum sensu stricto Strains and Its Implication for Diagnostic Purposes
Source: Front Microbiol. 2020 May 25;11:1002. doi: 10.3389/fmicb.2020.01002 (PMC7263005; doi:10.3389/fmicb.2020.01002)
Supplement: Supplementary file 6 [file Table_6.DOCX]

**Supplementary file 6a. Characteristics of introns and associated HEGs found in the *nad* genes**

| *nad1* | | | |  | *nad2* | | | | | | | | | | | | | *nad3* | | | | | |  | | *nad4L* | | | |  | *nad5* | | | |  |
| --- | --- | --- | --- | --- | --- | --- | --- | --- | --- | --- | --- | --- | --- | --- | --- | --- | --- | --- | --- | --- | --- | --- | --- | --- | --- | --- | --- | --- | --- | --- | --- | --- | --- | --- | --- |
|  | *i1* | *i2* |  |  |  | *i1* | |  | *i2* |  |  | |  |  | | |  |  | | *i1* | |  | |  | |  | *i1* |  | |  |  | *i1* |  | | *F. cerealis* |
|  |  | ○ |  |  |  | ● | |  | ● |  |  | |  |  | | |  |  | | ● | |  | |  | |  | ● |  | |  |  | ● |  | |  |
|  |  |  |  |  |  |  | |  |  |  |  | |  |  | | |  |  | |  | |  | |  | |  |  |  | |  |  |  |  | |  |
|  |  |  |  |  |  |  | |  |  |  |  | |  |  | | |  |  | |  | |  | |  | |  |  |  | |  |  |  |  | |  |
|  |  |  |  |  |  |  | |  |  |  |  | |  |  | | |  |  | |  | |  | |  | |  |  |  | |  |  |  |  | |  |
|  | *i1* | *i2* |  |  |  | *i1* | |  | *i2* |  | *i3* | |  | *i4* | | |  |  | | *i1* | |  | |  | |  | *i1* |  | |  |  | *i1* |  | | *F. culmorum* |
|  |  | ○ |  |  |  | ● | |  | ● |  | ● | |  | ● | | |  |  | | ● | |  | |  | |  | ● |  | |  |  | ● |  | |  |
|  |  |  |  |  |  |  | |  |  |  |  | |  |  | | |  |  | |  | |  | |  | |  |  |  | |  |  |  |  | |  |
|  |  |  |  |  |  |  | |  |  |  |  | |  |  | | |  |  | |  | |  | |  | |  |  |  | |  |  |  |  | |  |
|  |  |  |  |  |  |  | |  |  |  |  | |  |  | | |  |  | |  | |  | |  | |  |  |  | |  |  |  |  | |  |
|  | *i1* | *i2* |  |  |  | *i1* | |  | *i2* |  | *i3* | |  | *i4* | | |  |  | | *i1* | |  | |  | |  | *i1* |  | |  |  | *i1* |  | | *F. graminearum s.s.* |
|  |  | ○ |  |  |  | ● | |  | ● |  | ● | |  | ● | | |  |  | | ● | |  | |  | |  | ● |  | |  |  | ● |  | |  |
|  |  |  |  |  |  |  |  | |  |  |  |  | | |  |  | | |  | |  | |  | |  |  |  | |  |  |  |  | |  |  |
|  |  |  |  |  |  |  |  | |  |  |  |  | | |  |  | | |  | |  | |  | |  |  |  | |  |  |  |  | |  |  |
|  |  |  |  |  |  |  |  | |  |  |  |  | | |  |  | | |  | |  | |  | |  |  |  | |  |  |  |  | |  |  |
|  | *i1* | *i2* |  |  |  | *i1* |  | | *i2* |  | *i3* |  | | | *i4* |  | | |  | | *i1* | |  | |  |  | *i1* | |  |  |  | *i1* | |  | *F. pseudograminearum* |
|  |  | ○ |  |  |  | ● |  | | ● |  | ● |  | | | ● |  | | |  | | ● | |  | |  |  | ● | |  |  |  | ● | |  |  |

| Intron names: *i1 – i4* | | |  |  |  |  |
| --- | --- | --- | --- | --- | --- | --- |
| Intron type: | I | IA | IB | IC1 | IC2 | HEG type: ● - LAGLIDADG, ○ - GIY-YIG |

**Supplementary file 6b. Distribution of HEG homologs in the GenBank protein collection**

| Host | Intron and HEG | | | | | | | | | | | | |
| --- | --- | --- | --- | --- | --- | --- | --- | --- | --- | --- | --- | --- | --- |
|  | *nad1* | *nad2* | | | | |  | *nad3* |  | | *nad4L* |  | *nad5* |
|  | *i2* |  | *i1* | *i2* | *i3* | *i4* |  | *i1* | |  | *i1* |  | *i1* |
|  | ○ |  | ● | ● | ● | ● |  | ● | |  | ● |  | ● |
| *Fusarium cerealis* |  |  |  |  |  |  |  |  | |  |  |  |  |
| *Fusarium culmorum* |  |  |  |  |  |  |  |  | |  |  |  |  |
| *Fusarium graminearum s.s.* |  |  |  |  |  |  |  |  | |  |  |  |  |
| *Fusarium pseudograminearum* |  |  |  |  |  |  |  |  | |  |  |  |  |
| *Fusarium acuminatum* |  |  |  |  |  |  |  |  | |  |  |  |  |
| *Fusarium bambusae* |  |  |  |  |  |  |  |  | |  |  |  |  |
| *Fusarium circinatum* |  |  |  |  |  |  |  |  | |  |  |  |  |
| *Fusarium commune* |  |  |  |  |  |  |  |  | |  |  |  |  |
| *Fusarium gerlachii* |  |  |  |  |  |  |  |  | |  |  |  |  |
| *Fusarium oxysporum* |  |  |  |  |  |  |  |  | |  |  |  |  |
| *Fusarium temperatum* |  |  |  |  |  |  |  |  | |  |  |  |  |
| *Fusarium verticillioides* |  |  |  |  |  |  |  |  | |  |  |  |  |
| *Annulohypoxylon stygium* |  |  |  |  |  |  |  |  | |  |  |  |  |
| *Beauveria bassiana* |  |  |  |  |  |  |  |  | |  |  |  |  |
| *Bipolaris cookei* |  |  |  |  |  |  |  |  | |  |  |  |  |
| *Bipolaris maydis* |  |  |  |  |  |  |  |  | |  |  |  |  |
| *Bipolaris zeicola* |  |  |  |  |  |  |  |  | |  |  |  |  |
| *Botrytis cinerea* |  |  |  |  |  |  |  |  | |  |  |  |  |
| *Chrysoporthe austroafricana* |  |  |  |  |  |  |  |  | |  |  |  |  |
| *Coniothyrium glycines* |  |  |  |  |  |  |  |  | |  |  |  |  |
| *Cordyceps confragosa* |  |  |  |  |  |  |  |  | |  |  |  |  |
| *Cryphonectria parasitica* |  |  |  |  |  |  |  |  | |  |  |  |  |
| *Drechslerella brochopaga* |  |  |  |  |  |  |  |  | |  |  |  |  |
| *Handroanthus impetiginosus* |  |  |  |  |  |  |  |  | |  |  |  |  |
| *Hirsutella minnesotensis* |  |  |  |  |  |  |  |  | |  |  |  |  |
| *Hirsutella thompsoni* |  |  |  |  |  |  |  |  | |  |  |  |  |
| *Juglanconis juglandina* |  |  |  |  |  |  |  |  | |  |  |  |  |
| *Madurella mycetomatis* |  |  |  |  |  |  |  |  | |  |  |  |  |
| *Neurospora crassa* |  |  |  |  |  |  |  |  | |  |  |  |  |
| *Ophiocordyceps sinensis* |  |  |  |  |  |  |  |  | |  |  |  |  |
| *Pithomyces chartarum* |  |  |  |  |  |  |  |  | |  |  |  |  |
| *Podospora anserina* |  |  |  |  |  |  |  |  | |  |  |  |  |
| *Scytalidium sp.* |  |  |  |  |  |  |  |  | |  |  |  |  |
| *Sordaria macrospora* |  |  |  |  |  |  |  |  | |  |  |  |  |
| *Sporothrix brasiliensis* |  |  |  |  |  |  |  |  | |  |  |  |  |
| *Verticillium sp.* |  |  |  |  |  |  |  |  | |  |  |  |  |

| Identity | | |  |  |  |
| --- | --- | --- | --- | --- | --- |
| 90-100% | 80-90% | 70-80% | 60-70% |  |  |
|  |  |  |  |  |  |
| Hits were retained only if they had an e-value cut off lower than 0.001 and which covered at least 70% of the query sequence with >60% identity. | | | | |  |
| HEG type: ● - LAGLIDADG, ○ - GIY-YIG | | | | |  |
